# Enhanced prime editing systems by manipulating cellular determinants of editing outcomes

**DOI:** 10.1016/j.cell.2021.09.018

**Published:** 2021-10-28

**Authors:** Peter J. Chen, Jeffrey A. Hussmann, Jun Yan, Friederike Knipping, Purnima Ravisankar, Pin-Fang Chen, Cidi Chen, James W. Nelson, Gregory A. Newby, Mustafa Sahin, Mark J. Osborn, Jonathan S. Weissman, Britt Adamson, David R. Liu

**Affiliations:** 1 Merkin Institute of Transformative Technologies in Healthcare, Broad Institute of Harvard and MIT, Cambridge, MA 02141, USA; 2 Department of Chemistry and Chemical Biology, Harvard University, Cambridge, MA 02138, USA; 3 Howard Hughes Medical Institute, Harvard University, Cambridge, MA 02138, USA; 4 Department of Cellular and Molecular Pharmacology, University of California, San Francisco, San Francisco, CA 94158, USA; 5 Department of Microbiology and Immunology, University of California, San Francisco, San Francisco, CA 94158, USA; 6 Howard Hughes Medical Institute, University of California, San Francisco, San Francisco, CA 94158, USA; 7 Whitehead Institute for Biomedical Research, Massachusetts Institute of Technology, Cambridge, MA 02142, USA; 8 Department of Molecular Biology, Princeton University, Princeton, NJ 08544, USA; 9 Department of Pediatrics, University of Minnesota, Minneapolis, MN 55454, USA; 10 Center for Genome Engineering, University of Minnesota, Minneapolis, MN 55108, USA; 11 Stem Cell Institute, University of Minnesota, Minneapolis, MN 55455, USA; 12 Lewis-Sigler Institute for Integrative Genomics, Princeton University, Princeton, NJ 08544, USA; 13 Human Neuron Core, Rosamund Stone Zander Translational Neuroscience Center, Boston Children’s Hospital, Boston, MA 02115, USA; 14 Department of Neurology, Boston Children’s Hospital, Boston, MA 02115, USA; 15 Harvard Medical School, Boston, MA 02115, USA

**Keywords:** prime editing, genome editing, CRISPR-Cas9, Repair-seq, mismatch repair

## Abstract

While prime editing enables precise sequence changes in DNA, cellular determinants of prime editing remain poorly understood. Using pooled CRISPRi screens, we discovered that DNA mismatch repair (MMR) impedes prime editing and promotes undesired indel byproducts. We developed PE4 and PE5 prime editing systems in which transient expression of an engineered MMR-inhibiting protein enhances the efficiency of substitution, small insertion, and small deletion prime edits by an average 7.7-fold and 2.0-fold compared to PE2 and PE3 systems, respectively, while improving edit/indel ratios by 3.4-fold in MMR-proficient cell types. Strategic installation of silent mutations near the intended edit can enhance prime editing outcomes by evading MMR. Prime editor protein optimization resulted in a PEmax architecture that enhances editing efficacy by 2.8-fold on average in HeLa cells. These findings enrich our understanding of prime editing and establish prime editing systems that show substantial improvement across 191 edits in seven mammalian cell types.

## Highlights

• Pooled CRISPRi screens reveal that MMR inhibits prime editing efficiency and precision
• PE4 and PE5 enhance editing outcomes through co-expression of dominant negative MLH1
• Programming additional silent mutations can enhance prime editing by evading MMR
• PEmax editor improves prime editing efficacy in synergy with PE4, PE5, and epegRNAs

## Graphical abstract

**Figure:**
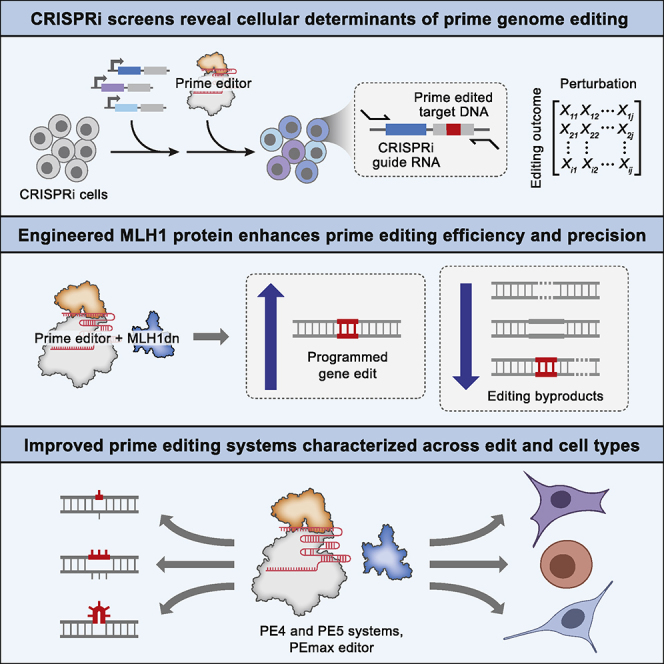

## Teaser

PE4 and PE5 are efficient and precise prime editing systems developed by leveraging insights into the way DNA repair pathways impact genome editing outcomes

## Introduction

The ability to manipulate the genome in a programmable manner has illuminated biology and shown promise in the clinical treatment of genetic diseases. Toward the goal of enabling a wide range of sequence changes, we developed prime editing, a versatile gene editing approach that can install all types of targeted DNA base pair substitutions, small insertions, small deletions, and combinations thereof without requiring double-strand DNA breaks (DSBs) or donor DNA templates (Anzalone et al., 2020; Anzalone et al., 2019). Prime editing has been broadly applied to introduce genetic changes in flies (Bosch et al., 2021), rice and wheat (Lin et al., 2020), zebrafish (Petri et al., 2021), mouse embryos (Liu et al., 2020), post-natal mice (Liu et al., 2021), human stem cells (Sürün et al., 2020), and patient-derived organoids (Schene et al., 2020). Despite its versatility, the efficiency of prime editing can vary widely across edit classes, target loci, and cell types (Anzalone et al., 2019). To maximize the utility of prime editing, we sought to identify cellular determinants of prime editing outcomes and use the resulting insights to develop improved prime editing systems.

**Figure 1:**
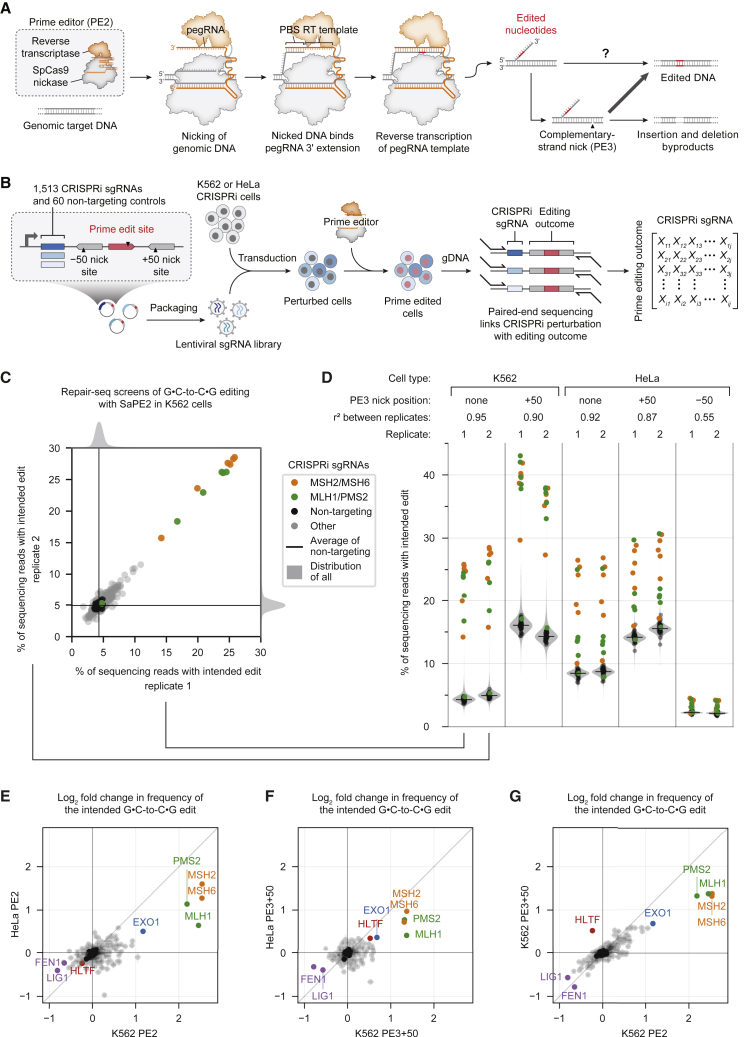
Pooled CRISPRi screens reveal genetic determinants of substitution prime editing outcomes (A) Prime editing with the PE2 system is mediated by the PE2 enzyme (*Streptococcus pyogenes* Cas9 [SpCas9] H840A nickase fused to an engineered reverse transcriptase) and a prime editing guide RNA (pegRNA). The PE3 system uses an additional single guide RNA (sgRNA) to nick the non-edited strand and yield higher editing efficiency. PBS, primer binding site; RT template, reverse transcription template. (B) Overview of prime editing Repair-seq screens. CRISPRi cells are transduced with a library of CRISPRi sgRNAs and a pre-validated prime edit site, then transfected with prime editors targeting the edit site. Paired-end sequencing of CRISPRi sgRNA identities and prime edited sites links each genetic perturbation with the associated editing outcome. (C) Effect of each CRISPRi sgRNA on the intended G⋅C-to-C⋅G prime edit at the targeted edit site in Repair-seq CRISPRi screens using PE2 in K562 cells. (D) Effect of CRISPRi sgRNAs on the intended edit in all screen conditions. Black dots represent individual non-targeting sgRNAs, black lines show the mean of all non-targeting sgRNAs, and gray shading represents kernel density estimates of the distributions of all sgRNAs. (E–G) Comparisons of gene-level effects of CRISPRi targeting on the intended G⋅C-to-C⋅G prime edit across different screen conditions. (E) K562 PE2 versus HeLa PE2. (F) K562 PE3+50 versus HeLa PE3+50. (G) K562 PE2 versus K562 PE3+50. The effect of each gene is calculated as the average log_2_ fold change in frequency from non-targeting sgRNAs for the two most extreme sgRNAs targeting the gene. Dots represent the mean of n = 2 independent replicates for each cell type, and bars show the range of values spanned by the replicates. Black dots represent 20 random sets of three non-targeting sgRNAs.

Prime editing minimally requires two components: an engineered reverse transcriptase (RT) fused to Cas9 nickase (the PE2 protein) and a prime editing guide RNA (pegRNA) that contains both a spacer sequence complementary to target DNA and a 3′ extension encoding the desired edit (Anzalone et al., 2019) (Figure 1A). The PE2–pegRNA complex binds one strand of a target DNA locus and nicks the opposite strand, exposing a DNA 3′ end that can hybridize to the primer binding site (PBS) in the pegRNA extension. Reverse transcription of the RT template within the pegRNA extension then generates a 3′ DNA flap that contains the edited sequence and ultimately leads to incorporation of that sequence into the genome. The “PE3” system differs from PE2 by using an additional single guide RNA (sgRNA) to nick the non-edited strand at a location away from the pegRNA target, which enhances editing efficiency. However, nicking the non-edited strand also increases the frequency of undesired insertions and deletions (indels) at the target site.

**Figure S1:**
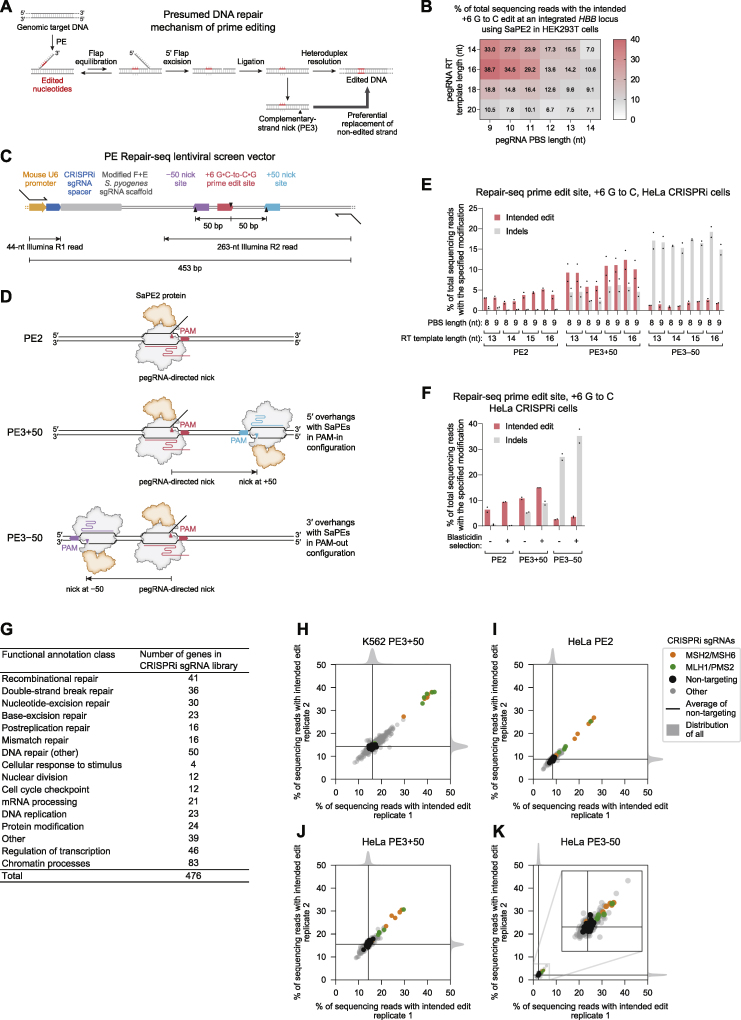
Design and results of Repair-seq screens for substitution prime editing outcomes, related to Figure 1 (A) Presumed model by which the reverse-transcribed 3′ DNA flap is permanently incorporated into the genome during prime editing (Anzalone et al., 2019). (B) Installation of a G⋅C-to-C⋅G edit within a lentivirally integrated *HBB* sequence using SaPE2 and Sa-pegRNAs in HEK293T cells. PBS, primer binding site. Data represent the mean of n = 3 independent replicates. (C) Design of the prime editing Repair-seq lentiviral vector (pPC1000, additional details and full sequence information in STAR Methods). In Repair-seq screens, a 453-bp region containing CRISPRi sgRNA sequence and prime editing outcome is amplified from genomic DNA for paired-end Illumina sequencing. The CRISPRi sgRNA is sequenced with a 44-nt Illumina forward read (R1), and the prime edited site (including +50 and –50 nick sites) is sequenced with a 263-nt Illumina reverse read (R2). Black triangles indicate positions of SaPE2-induced nicks programmed by Sa-pegRNA and Sa-sgRNAs. Sizes of all vector components are to scale. (D) Schematic of PE2, PE3+50, and PE3–50 prime editing configurations with SaPE2 protein (SaCas9 N580A fused to an engineered MMLV RT). (E) Validation of intended G⋅C-to-C⋅G editing at the lentivirally integrated Repair-seq edit site in HeLa cells expressing dCas9–BFP–KRAB cells. Bars represent the mean of n = 2 independent replicates. (F) Prime editing at the Repair-seq edit site with blasticidin selection in HeLa cells expressing dCas9–BFP–KRAB. SaPE2–P2A–BlastR prime editor was used for all conditions. Bars represent the mean of n = 2 independent replicates. (G) Functional annotation classes of the genes targeted by the pooled CRISPRi sgRNA library used in Repair-seq screens. (H–K) Knockdown of *MSH2*, *MSH6*, *MLH1*, and *PMS2* increases the frequency of the intended +6 G⋅C-to-C⋅G prime edit in all Repair-seq screens. Dots represent individual CRISPRi sgRNAs.

*In vitro* experiments provide support for the early steps of prime editing (Anzalone et al., 2019), but mechanisms downstream of 3′ flap synthesis remain speculative. According to the current model, the newly synthesized 3′ flap displaces an adjacent strand of genomic DNA through flap interconversion (Figure S1A). Excision of the displaced 5′ flap then allows ligation of the edited sequence into the genome. Nicking the non-edited strand in the PE3 system is thought to induce cellular replacement of the non-edited strand and thus promote installation of the edited sequence in both strands.

The presumed involvement of cellular factors in these steps motivated us to study the roles of DNA repair mechanisms in prime editing and to develop improved prime editing systems through manipulation of those processes. Here, we used pooled CRISPR interference (CRISPRi)-based screens to systematically probe the effect of 476 genes involved in DNA repair and associated processes on substitution prime editing outcomes. We discovered that specific DNA mismatch repair (MMR) genes strongly suppress prime editing efficiency and promote indel formation. Consistent with a model in which MMR reverts heteroduplex DNA formed during prime editing, we identified classes of prime edits that are less vulnerable to MMR activity and are therefore generated more efficiently. Integrating these findings, we developed improved prime editing systems through transient expression of a dominant negative MMR protein (MLH1dn). In six MMR-proficient cell types, including induced pluripotent stem cells (iPSCs) and primary T cells, these PE4 (PE2+MLH1dn) and PE5 (PE3+MLH1dn) systems enhanced editing efficiency over PE2 and PE3 by an average of 7.7-fold and 2.0-fold, respectively, and increased edit/indel ratios (outcome purity) by 3.4-fold. Transient co-expression of MLH1dn did not result in detected changes to microsatellite repeat length, a clinical biomarker of MMR proficiency (Umar et al., 2004). Strategic installation of additional silent mutations nearby an intended edit can also improve prime editing efficiency by evading MMR recognition, even in the absence of MLH1dn. Finally, we engineered an optimized “PEmax” prime editor architecture that further increased editing efficiency in synergy with PE4, PE5, and engineered pegRNAs (epegRNAs) (Nelson et al., 2021). These findings deepen our understanding of prime editing and establish prime editing systems with substantially improved efficiency and outcome purity across 191 different edits at 20 loci in seven mammalian cell types.

## Results

### Design of a pooled CRISPRi screen for prime editing outcomes

We reasoned that identifying genetic determinants of prime editing sequence outcomes, including the original sequence, the desired edit, and indels, could inform strategies to maximize efficiency and minimize unwanted byproducts. We therefore used a genetic screening approach to study prime editing. This method, called Repair-seq, measures the effects of many loss-of-function perturbations on the outcomes of genome editing experiments by linking the identity of CRISPRi sgRNAs to edited sites in pooled screens (Hussmann et al., 2021) (Figure 1B). Briefly, a library of sgRNAs are transduced into cells expressing the CRISPRi effector (dCas9–KRAB) such that most cells receive only one sgRNA, causing the knockdown of one gene per cell. After genome editing occurs at an adjacent target site delivered on the same lentiviral cassette, paired-end sequencing enables the frequency of each editing outcome to be measured for each linked CRISPRi perturbation.

To enable Repair-seq screens for prime editing outcomes, we first made prime editing and CRISPRi orthogonal. Typically, these systems both rely on *Streptococcus pyogenes* Cas9 (SpCas9). We constructed an SaPE2 prime editor variant by replacing the SpCas9 nickase domain in PE2 with *Staphylococcus aureus* Cas9 (SaCas9) N580A nickase (Ran et al., 2015) and verified SaPE2 editing activity with orthogonal *S. aureus* pegRNAs (Sa-pegRNAs) (Figure S1B). Next, we built a Repair-seq vector for screening with a composite SaPE2 edit site. This site comprised a target protospacer that is efficiently prime edited in HEK293T cells (Figure S1B) and two flanking protospacers that allow complementary-strand nicks 50 bp downstream (+50 nick) or upstream (−50 nick) of the target (Figures 1B and S1C). This design supports SaPE2 prime editing in three configurations: PE2, PE3 with a +50 nick (PE3+50), or PE3 with a −50 nick (PE3−50) (Figure S1D). In a validated HeLa CRISPRi cell line (Gilbert et al., 2013), we observed G⋅C-to-C⋅G prime editing efficiencies up to 9.4% with PE2, 15% with PE3+50, and 3.5% with PE3−50 (Figures S1E and S1F) at this composite edit site, establishing a screening assay well suited to detect increases or decreases in editing from CRISPRi perturbations.

### Identification of DNA repair genes that affect prime editing outcomes

We performed Repair-seq screens of prime editing outcomes with PE2 and PE3+50 in K562 and HeLa cells and with PE3−50 in HeLa cells. We transduced a Repair-seq library of 1,513 sgRNAs targeting 476 genes (enriched for roles in DNA repair and associated processes; Figure S1G) and 60 non-targeting control sgRNAs into human K562 and HeLa CRISPRi cell lines (Gilbert et al., 2014; Gilbert et al., 2013; Hussmann et al., 2021) (Figure 1B; Table S1). Next, we transfected these cells with SaPE2, Sa-pegRNA, and Sa-sgRNA plasmids that program a G⋅C-to-C⋅G transversion at the co-transduced edit site. Finally, we extracted genomic DNA, amplified the CRISPRi sgRNA and edit site by PCR, and performed paired-end sequencing to measure the distribution of editing outcomes for each genetic perturbation (Figure S1C). To interpret the resulting data, we compared the frequencies of editing outcomes from cells containing a gene-targeting CRISPRi sgRNA to the corresponding frequencies from cells containing non-targeting sgRNA controls. Reduction in an outcome’s frequency upon gene knockdown suggests that the gene’s activity promotes formation of the outcome, while an increase in frequency suggests that the gene’s activity suppresses the outcome.

We first examined the effect of gene knockdowns on the frequency of the intended G⋅C-to-C⋅G edit. In cells with non-targeting CRISPRi sgRNAs, 4.3%–4.9% (K562) and 8.5%–8.7% (HeLa) of sequencing reads contained exactly the intended edit following PE2 editing (Figures 1C and 1D). These levels increased to 14%–16% (K562) and 14%–16% (HeLa) for PE3+50 but decreased to 2.1%–2.2% (HeLa) for PE3−50. Across all screen conditions, CRISPRi targeting of *MSH2*, *MSH6*, *MLH1*, and *PMS2*, components of the MutSα–MutLα MMR complex (Iyer et al., 2006; Kunkel and Erie, 2005; Li, 2008), substantially increased editing efficiency by up to 5.8-fold for PE2, 2.5-fold for PE3+50, and 2.0-fold for PE3−50 (Figures 1E–1G and S1H–S1K; Table S2). Knockdown of *EXO1*, an exonuclease with a role in MMR (Genschel et al., 2002), also increased intended PE2 editing efficiency by up to 2.3-fold. In contrast, knockdown of *LIG1*, a nick-sealing DNA ligase (Pascal et al., 2004), and of *FEN1*, a 5′ flap endonuclease (Liu et al., 2004), reduced the frequency of intended editing, consistent with their previously proposed roles in nick ligation and 5′ flap excision during prime editing (Anzalone et al., 2019). Together, these data suggest that MMR activity antagonizes the installation of point mutations by prime editing.

**Figure 2:**
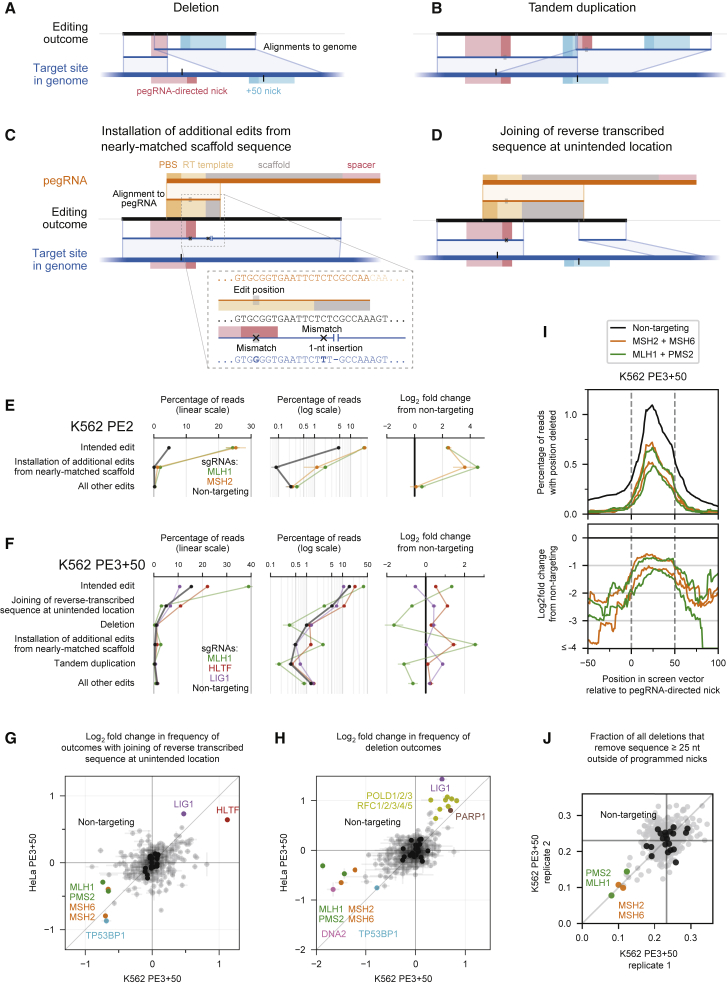
Genetic modulators of unintended prime editing outcomes (A–D) Representative examples of four categories of unintended prime editing outcomes observed in CRISPRi screens. Blue and orange lines between the editing outcome and the genome or pegRNA depict local sequence alignments. X’s represent mismatches in alignments, gaps represent insertions, and gray boxes represent the location of the programmed edit. Red and cyan rectangles on the genome mark SaCas9 protospacers and PAMs, and black vertical lines mark the locations of SaCas9 nick sites. (E and F) Summary of editing outcome categories observed in PE2 screens (E) and in PE3+50 screens (F) in K562 cells. Plotted quantities are the mean ± SD of all sgRNAs for each indicated gene (60 non-targeting sgRNAs and three sgRNAs per targeted gene), averaged across n = 2 independent replicates. (G and H) Comparison of the effects of knockdown of all genes targeted in CRISPRi screens on the frequency of joining of reverse-transcribed sequence at unintended location (G) or of deletions (H) from PE3+50. The effect of each gene is calculated as the average log_2_ fold change in frequency from non-targeting sgRNAs for the two most extreme sgRNAs targeting the gene. Dots represent the mean of n = 2 independent replicates for each cell type, and bars show the range of values spanned by the replicates. Black dots represent 20 random sets of three non-targeting sgRNAs. (I) Top: frequency of deletion as a function of genomic position relative to programmed PE3+50 nicks (dashed vertical lines) in K562 screen replicate 1 across all reads for indicated sets of CRISPRi sgRNAs (black line, 60 non-targeting sgRNAs; orange and green lines, three sgRNAs targeting each of *MSH2*, *MSH6*, *MLH1*, and *PMS2*). Bottom: log_2_ fold change in frequency of deletion as a function of genomic position for *MSH2*, *MSH6*, *MLH1*, and *PMS2* sgRNAs compared to non-targeting sgRNAs. (J) Effect of gene knockdowns on the fraction of all observed deletions that remove sequence at least 25 nt outside of programmed PE3+50 nicks in K562 screens. Each dot represents all reads for sgRNAs targeting an individual gene. Black dots represent 20 sets of three random non-targeting sgRNAs.

**Figure S2:**
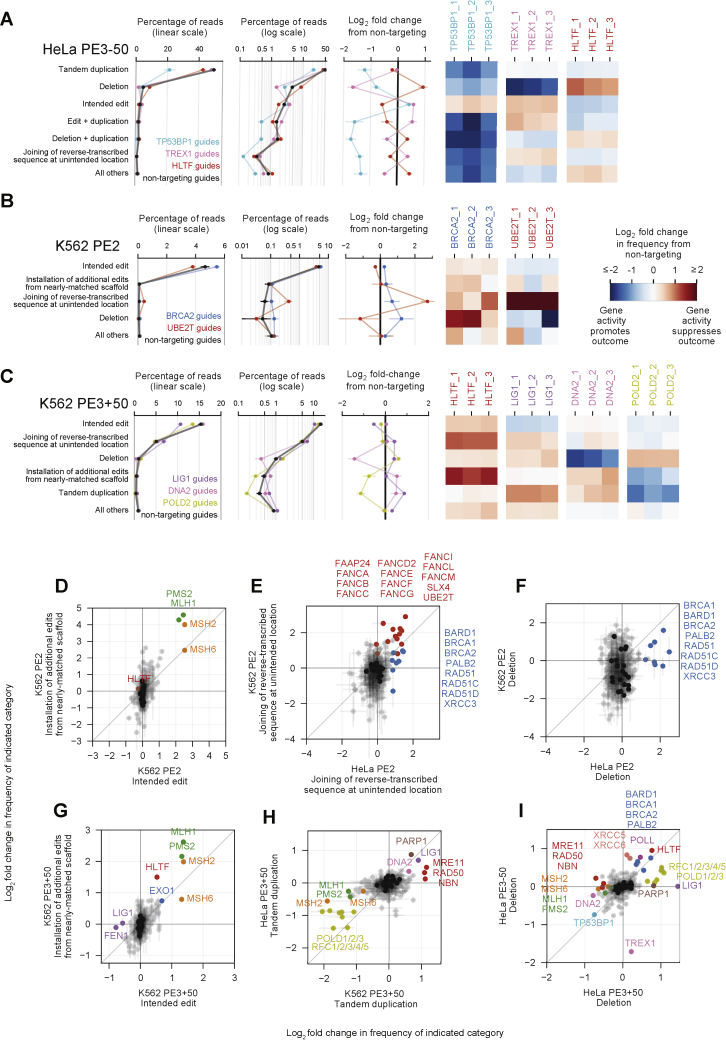
Genetic modulators of unintended prime editing outcomes, related to Figure 2 (A) Overview of PE3–50 outcomes in HeLa CRISPRi screens. *TP53BP1* knockdown dramatically reduces formation of all unintended editing outcomes. (B) Additional details of PE2 outcomes in K562 CRISPRi screens, supplementing Figure 2E. (C) Additional details of PE3+50 outcomes in K562 CRISPRi screens, supplementing information in Figure 2F. (D–I) Comparisons of effects of gene knockdown on frequencies of indicated outcome categories in indicated screen conditions. Plotted quantities are the mean of the log_2_ fold changes from non-targeting sgRNAs for the two most extreme sgRNAs per gene, averaged over n = 2 independent replicates per condition. Error bars mark the range of values spanned by the replicates. Black dots represent 20 random sets of three non-targeting sgRNAs. (D) *MSH2*, *MLH1*, and *PMS2* knockdown produce larger fold changes in installation of additional edits than in intended edits in K562 PE2 screens. (E) Unintended joining of reverse transcribed sequence in PE2 screens in K562 and HeLa cells are most increased by knockdown of Fanconi anemia genes (red) as well as a set of *RAD51* homologs and other genes involved in homologous recombination (blue). (F) Deletions in PE2 screens in K562 and HeLa cells are most increased by a set of *RAD51* homologs and other genes involved in homologous recombination (blue). (G) In addition to *MSH2*, *MLH1*, and *PMS2*, *HLTF* knockdown produces larger fold changes in installation of additional edits than in intended edits in K562 PE3+50 screens. (H) Tandem duplications in HeLa and K562 PE3+50 screens are most decreased by knockdown of POLD and RFC subunits. (I) Deletions in HeLa PE3+50 and PE3–50 screens have dramatically divergent genetic regulators, highlighting differences in the processing of the different overhang configurations.

In addition to the intended edit, Repair-seq screens identified four primary categories of editing byproducts: deletions (Figure 2A), tandem duplications (Figure 2B), and two classes of outcomes containing unintended sequence from the pegRNA (Figures 2C and 2D). We observed low baseline frequencies of total unintended edits from PE2 (0.31% in K562, 0.60% in HeLa; Figure 2E) but more frequent and diverse unintended byproducts from PE3−50 (58% in HeLa; Figure S2A) and PE3+50 (8.2% in K562, 9.5% in HeLa; Figure 2F). The baseline frequencies and genetic modulators of these categories varied across PE2, PE3+50, and PE3−50 screens, providing a rich set of observations of how different prime editing configurations are processed (Figures S2A–S2I).

**Figure S3:**
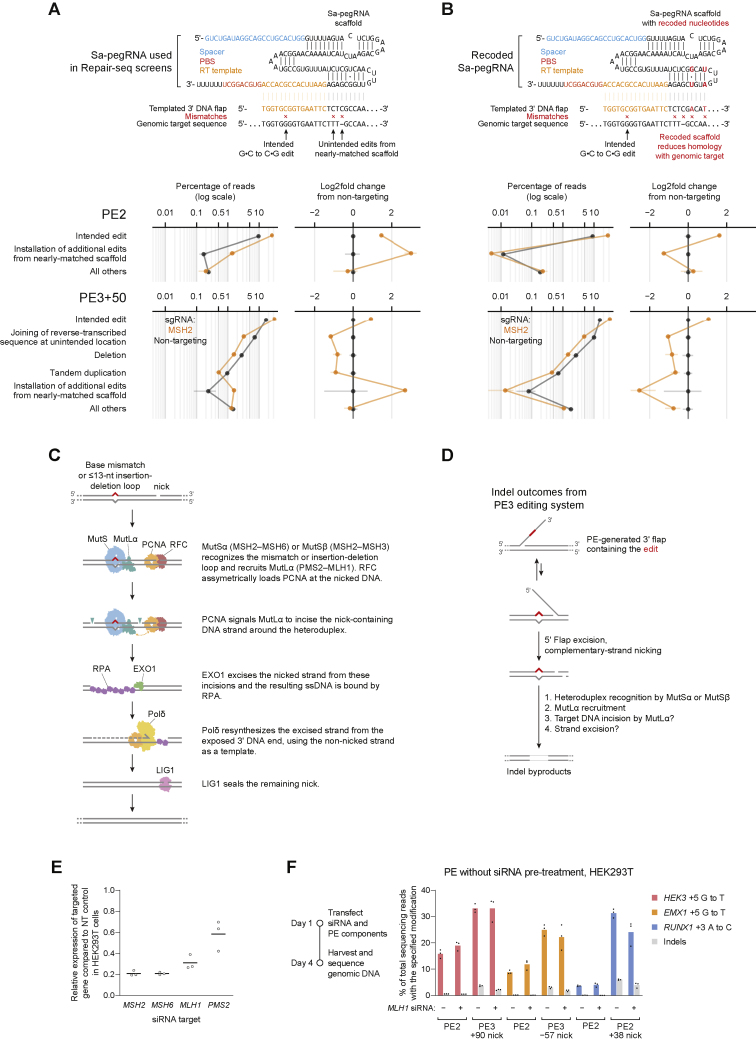
Validation of prime editing Repair-seq screen results, related to Figures 2 and 3 (A–B) Top: alignment of Sa-pegRNAs, their templated 3′ DNA flaps following SaPE2 reverse transcription, and the genomic target sequence. Compared to the Sa-pegRNA used in Repair-seq screens (A), an Sa-pegRNA with recoded scaffold sequence (B) templates an extended 3′ DNA flap with reduced homology with genomic target sequence. The recoded Sa-pegRNA contains 2 base pair changes that preserve base pairing interactions within the scaffold. Reverse transcription of the Sa-pegRNA scaffold can generate a misextended 3′ flap that is incorporated into the genome. Vertical lines depict base pairing. Red X’s depict mismatches between the misextended reverse-trancribed 3′ flap and genomic sequence. Bottom: frequencies of editing outcome categories observed at the screen edit site from arrayed PE2 and PE3+50 experiments in HeLa CRISPRi cells. Prime editing with the Sa-pegRNA used in Repair-seq screens (A) or a recoded Sa-pegRNA (B) results in different frequencies of installation of unintended edits from nearly matched scaffold. Plotted quantities are the mean ± SD of n = 4 independent replicates for cells containing a *MSH2* or non-targeting CRISPRi sgRNA. (C) Mechanism of DNA mismatch repair in humans. (D) Mismatch repair of a prime editing heteroduplex intermediate induces indel byproducts, potentially through MutLα endonuclease activity at the target locus or excision from these non-programmed nicks and subsequent repair of the resulting intermediates. (E) Knockdown efficiency of siRNA treatment relative to a non-targeting siRNA control in HEK293T cells. Cells were transfected with siRNAs, incubated for 3 days, transfected with PE2, pegRNAs, and the same siRNAs, then incubated for another 3 days before relative RNA abundances were assayed by RT-qPCR. NT, non-targeting. Data represent the mean of n = 3 independent replicates. Each dot represents the mean of n = 3 technical replicates. Data supplements information in Figure 3C. (F) Editing in HEK293T cells co-transfected with prime editor components and siRNAs. Cells were not pre-treated with siRNAs before transfection with prime editors. Bars represent the mean of n = 3 independent replicates.

Two of these observations informed models for the role of MMR activity during prime editing. First, one unintended outcome contained the intended G⋅C-to-C⋅G edit as well as an additional base substitution and a 1-nt insertion near the target site (Figure 2C). The sequence around these additional mutations perfectly matched 9 nt at the 3′ end of the pegRNA scaffold sequence, consistent with reverse transcription into the pegRNA scaffold and incorporation of the resulting 3′ DNA flap into partially homologous genomic sequence. Recoding the pegRNA scaffold to avoid sequence homology with the genomic target reduced the frequency of this outcome category (Figures S3A and S3B), suggesting a general approach to eliminate this class of editing byproduct. Notably, we observed that knockdown of MMR genes increased the frequency of this editing byproduct from 0.08% to 2.0% in PE2 K562 screens (Figures 2E and S2D). MMR thus suppresses the formation of this outcome to a larger extent than the intended edit, indicating that distinct prime editing intermediates can differ in the extent to which they are processed by MMR.

Second, MMR knockdown reduced the frequency of most categories of unintended outcomes from PE3+50 (Figures 2F–2H, S2H, and S2I), suggesting that transiently inhibiting some MMR activities may increase both the efficiency and outcome purity of prime editing. The most abundant class of unintended PE3+50 outcomes contained sequence from the reverse-transcribed 3′ DNA flap that does not rejoin genomic sequence at the intended flap annealing location (5.1% of non-targeting reads in K562, 3.8% in HeLa; Figure 2D). In both cell types, knockdown of MMR genes reduced the frequency of unintended flap rejoining outcomes, by up to 1.7-fold (Figure 2G). Similarly, MMR knockdown substantially reduced deletions from PE3+50 by up to 3.7-fold (Figure 2H). Intriguingly, genomic sequence between the two SaPE2-induced nicks was most frequently deleted for PE3+50, but MMR knockdown in K562 cells decreased the frequency of deletions outside of these programmed nicks to a greater extent than deletions between them (Figures 2I and 2J), suggesting that MMR activity may generate longer deletion byproducts during prime editing. Finally, tandem duplications of sequence between the nicks, which were common for PE3−50 (Figure S2A) but rarer for PE3+50 (0.37% of non-targeting reads in K562, 2.3% in HeLa), were reduced by up to 3.7-fold (K562) and 1.5-fold (HeLa) by MMR knockdown (Figures 2F and S2H).

### Model for MMR of prime editing intermediates

The effects of MMR knockdown in these Repair-seq screens led to a working model for the role of MMR during prime editing. In eukaryotes, MMR resolves DNA heteroduplexes containing a base mismatch or small insertion-deletion loop (IDL) by selectively replacing nicked DNA strands (Iyer et al., 2006; Kunkel and Erie, 2005; Li, 2008) (Figure S3C). To initiate MMR, the heteroduplex is first bound by MutSα (MSH2–MSH6) or MutSβ (MSH2–MSH3), which recognize base mismatches and IDLs less than 13 nt in length (Gupta et al., 2011; Warren et al., 2007). Next, MSH2 recruits MutLα (PMS2–MLH1), which incises the nick-containing strand around the heteroduplex (Fang and Modrich, 1993; Kadyrov et al., 2006; Pluciennik et al., 2010; Thomas et al., 1991). Finally, EXO1 excises the heteroduplex from these incisions (Genschel et al., 2002), polymerase δ resynthesizes the excised DNA strand, and ligase I (LIG1) seals the nascent strand (Zhang et al., 2005).

**Figure 3:**
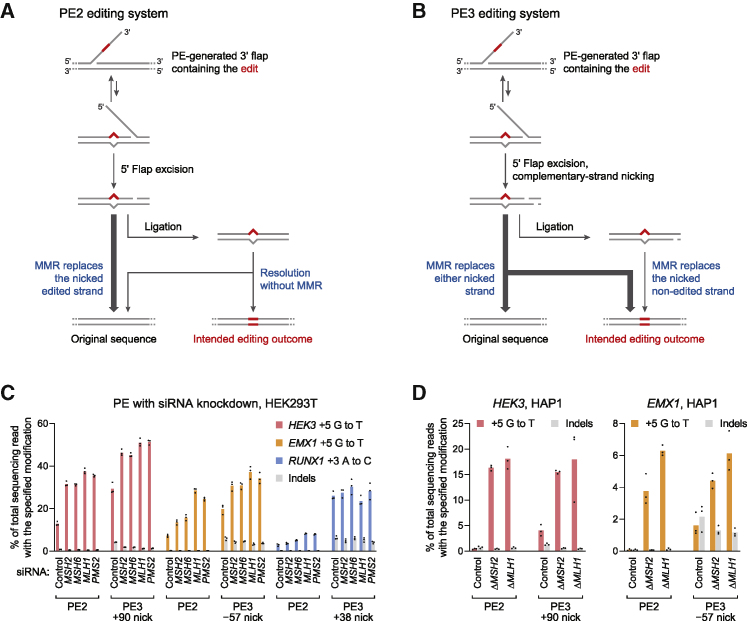
Model for mismatch repair of prime editing intermediates (A) Model for DNA mismatch repair (MMR) of PE2 intermediates. MMR replaces the nicked strand during repair of the heteroduplex PE intermediate. Ligation of the nick before MMR recognition removes the strand discrimination signal for MMR, resulting in unbiased resolution of the heteroduplex. (B) Model for MMR of PE3 intermediates. Nicks on both DNA strands can direct MMR to replace either strand. Ligation of the nick on the edited strand would guide MMR to replace the non-edited strand. (C) Prime editing at endogenous sites in HEK293T cells pretreated with siRNAs (details in STAR Methods). Bars represent the mean of n = 3 independent replicates. (D) Prime editing in HAP1 *ΔMSH2* and HAP1 *ΔMLH1* cells. Δ, gene knockout. Bars represent the mean of n = 3 independent replicates.

We hypothesized that MMR engages a specific prime editing intermediate, a DNA heteroduplex formed by hybridization of the reverse-transcribed 3′ DNA flap to adjacent genomic DNA (Figure 3A). MutSα or MutSβ may recognize the heteroduplex within this structure, and the 3′ nick present after flap equilibration, but before ligation, could stimulate selective excision of the edited strand and subsequent repair to regenerate the original, unedited sequence. Alternatively, MMR may prevent productive flap interconversion by rejecting annealing of the edited 3′ flap to the genomic target (Sugawara et al., 2004). In either case, inhibiting MMR during prime editing could delay heteroduplex repair or increase the likelihood of nick ligation, removing bias in the repair of the edited product. Consistent with this model, knockdown of MutSα–MutLα genes strongly enhanced PE2 editing by up to 5.8-fold (Figures 1C and 1D). Interfering with MMR reversion of these intermediates can thus enhance prime editing efficiency.

In the context of PE3, nicking the non-edited strand of the heteroduplex intermediate may direct MMR to more frequently replace that strand, leading to higher prime editing efficiency and dampened benefits of MMR suppression, as we observed (Figures 1G and 3B). Nevertheless, the detrimental overall effect of MMR activity on PE3 suggests that heteroduplex intermediates that favorably bias MMR toward the desired edit—those with a ligated edited strand and a nicked non-edited strand—are uncommon. In addition to increasing the intended prime edit, knockdown of MMR genes also reduced indel byproducts from PE3+50 (Figure 2F). Given this observation, we hypothesize that during repair of a prime editing heteroduplex, MMR activity may induce DSBs, possibly via nicking or excision of the target locus (Figure S3D). In agreement with this hypothesis, knockdown of MutSα–MutLα genes disproportionately reduced PE3+50 deletion outcomes outside of the sequence between pegRNA and sgRNA nicks (Figure 2I). Altogether, these findings support a model in which MMR activity strongly suppresses intended prime editing outcomes and instead promotes indel byproducts.

### MMR inhibition improves prime editing at endogenous loci

To validate the above model, we tested the effect of MMR on prime editing with canonical SpCas9-based prime editors at endogenous genomic loci and in additional cell types. We treated HEK293T cells with small interfering RNA (siRNAs) targeting MutSα and MutLα genes, cultured the cells for 3 days to allow siRNA-mediated knockdown (Figure S3E), and then transfected plasmids encoding PE2 and pegRNAs that program point mutations. Across three sites, we observed that mRNA knockdown strongly increased average PE2 editing from 7.7% to 25% with a decrease in indel frequency from 0.39% to 0.28% (Figure 3C) but improved average PE3 editing efficiency to a lesser extent (from 25% to 37%). Additionally, knockdown of MMR genes reduced the frequency of PE3 indels from 5.5% down to 3.2% on average, increasing PE3 outcome purity by 2.9-fold (Figure 3C). Thus, consistent with our model, the impact of MMR on PE3 editing efficiency is tempered by its opposing effects on reverting the 3′ flap intermediate (which impedes prime editing) and mediating replacement of the unedited strand (which promotes prime editing).

We also measured prime editing in MMR-deficient Δ*MSH2* or Δ*MLH1* haploid HAP1 cells. PE2 editing was much more efficient in MMR-deficient cells (17% at *HEK3* and 5.0% at *EMX1*) than in wild-type control cells (0.44% at *HEK3,* 0.07% at *EMX1*; Figure 3D). However, complementary-strand nicking did not affect prime editing efficiency in MMR-deficient cells (Figure 3D), consistent with our model that complementary-strand nicking improves prime editing by influencing MMR strand selectivity. Taken together, these results further support a model in which MMR impedes prime editing by promoting excision of the edited DNA strand, even though this effect is partially counterbalanced in the PE3 system by the role of MMR in replacing the non-edited strand.

### Engineered dominant negative MLH1 enhances prime editing efficiency and precision

**Figure 4:**
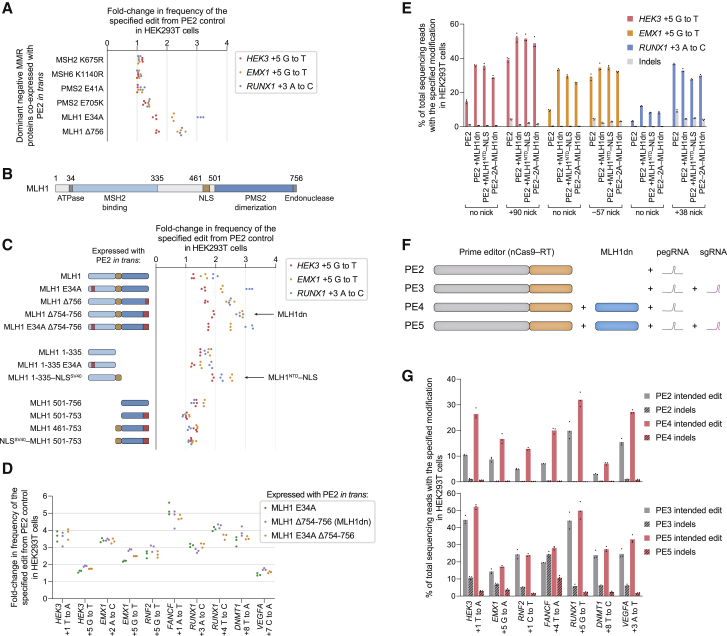
Engineered dominant negative MLH1 enhances prime editing outcomes (A) Co-expression of PE2 with dominant negative variants of human MMR proteins improves prime editing efficiency. All values from n = 3 independent replicates are shown. (B) Functional annotation of the 756-aa human MLH1 protein. (C) Editing enhancement from MLH1 variants co-expressed with PE2. Red boxes indicate mutations that inactivate MLH1 ATPase or endonuclease function. All values from n = 3 independent replicates are shown. (D) Comparison of the top three MLH1 variants across ten prime edits. All values from n = 3 independent replicates are shown. (E) Prime editing with PE2 and MLH1dn in *trans*, PE2 and MLH1^NTD^–NLS in *trans*, and PE2–P2A–MLH1dn (human codon-optimized). Bars represent the mean of n = 3 independent replicates. (F) The PE4 editing system consists of a prime editor enzyme (nickase Cas9–RT fusion), MLH1dn, and pegRNA. The PE5 editing system consists of a prime editor enzyme, MLH1dn, pegRNA, and nicking sgRNA. (G) PE2, PE3, PE4, and PE5 editing in HEK293T cells. Bars represent the mean of n = 3 independent replicates.

**Figure S4:**
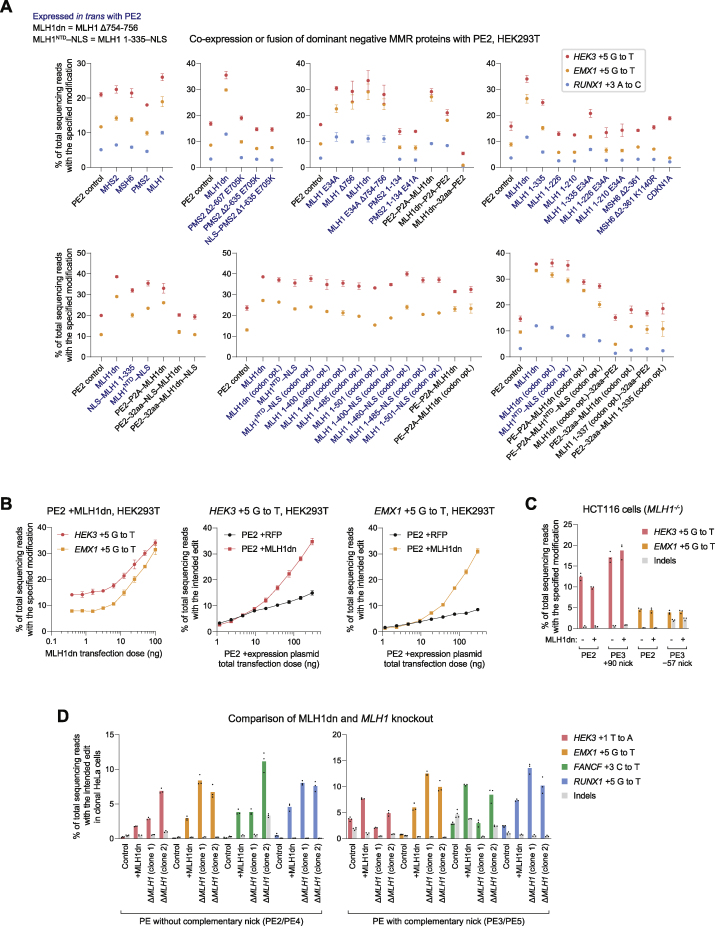

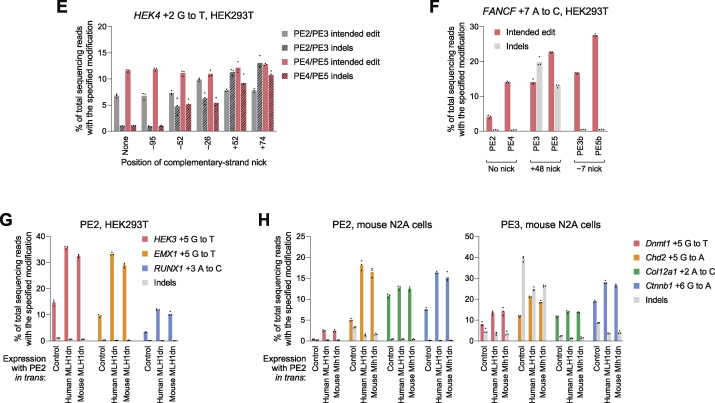
Development and characterization of dominant negative MMR proteins that enhance prime editing outcomes, related to Figure 4 (A) Prime editing efficiencies from MMR proteins or dominant negative variants expressed *in trans* with or fused directly to PE2 in HEK293T cells. 32aa, (SGGS)×2–XTEN16–(SGGS)×2 linker. codon opt., human codon-optimized. Data within the same graph originate from experiments performed at the same time. Data represent the mean ± SD of n = 3 independent replicates. (B) Titration of MLH1dn plasmid and PE2 plasmid transfection doses in HEK293T cells. Maximum plasmid amounts tested were 200 ng PE2 and 100 ng MLH1dn. Data represent the mean ± SD of n = 3 independent replicates. (C) Prime editing with MLH1dn co-expression in MMR-deficient HCT116 cells that contain a biallelic deletion in *MLH1*. Bars represent the mean of n = 3 independent replicates. (D) *MLH1* knockout in clonal HeLa cell lines enhances prime editing efficiency to a greater extent than MLH1dn co-expression in clonal wild-type HeLa cells. Δ, knockout. Bars represent the mean of n = 3 or 4 independent replicates. (E) Editing at the *HEK4* locus with complementary-strand nicks in HEK293T cells. “None” indicates the lack of a nick, which denotes a PE2 or PE4 editing strategy. Bars represent the mean of n = 3 independent replicates. (F) Editing at the *FANCF* locus with PE3b and PE5b (complementary-strand nick that is specific for the edited sequence) in HEK293T cells. PE5b, PE3b editing system with MLH1dn co-expression. Bars represent the mean of n = 3 independent replicates. (G) Comparison of prime editing with human MLH1dn (human codon-optimized) or mouse MLH1dn (mouse codon-optimized) in human HEK293T cells. Bars represent the mean of n = 3 independent replicates. (H) Comparison of prime editing with human MLH1dn (human codon-optimized) or mouse MLH1dn (mouse codon-optimized) in mouse N2A cells. Bars represent the mean of n = 3 independent replicates.

Encouraged that cellular pretreatment with MMR-targeting siRNAs could enhance prime editing efficiency, we next explored strategies for simultaneous co-delivery of prime editors and MMR-inhibiting agents. Co-transfection of PE2 and *MLH1* siRNAs without pretreatment did not substantially increase editing efficiency after 3 days (Figure S3F), as expected given the kinetics of RNA silencing (Bartlett and Davis, 2006). We hypothesized that dominant negative MMR protein variants could instead be transiently co-expressed with PE2 or as fusions with PE2 to enhance prime editing. We co-transfected HEK293T cells with plasmids encoding PE2, pegRNAs, and catalytically impaired mutants of human MSH2, MSH6, PMS2, and MLH1 (Gueneau et al., 2013; Iaccarino et al., 1998; Kadyrov et al., 2006; Räschle et al., 2002; Tomer et al., 2002) (Figures 4A). Of these mutants, ATPase-impaired MLH1 E34A and endonuclease-impaired MLH1 Δ756 increased PE2 editing efficiency by 1.6- to 3.1-fold for three substitution prime edits. Testing additional MLH1 variants, we observed that a larger endonuclease-impaired MLH1 deletion (MLH1 Δ754–756) enhanced average PE2 editing efficiency to the greatest extent across 10 edits (3.2-fold), but combining ATPase and endonuclease mutations (MLH1 E34A Δ754–756) did not yield additional improvement (Figures 4B–4D and S4A). We therefore designated MLH1 Δ754–756 as MLH1dn.

We also identified shorter MLH1 truncations that can inhibit MMR during prime editing. The MLH1 N-terminal domain (NTD; residues 1–335) mediates MutLα recruitment to MSH2 during MMR (Plotz et al., 2003), while the MLH1 C-terminal domain (CTD; residues 501–756) dimerizes with PMS2 and contributes to MutLα endonuclease activity critical for MMR (Gueneau et al., 2013; Guerrette et al., 1999) (Figure 4B). The MLH1 NTD with a nuclear localization signal (NLS) tag (hereafter referred to as MLH1^NTD^–NLS) improved PE2 editing by 1.9- to 2.5-fold (Figures 4C and S4A), to a similar degree as full-length MLH1dn. In contrast, the MLH1 CTD did not substantially enhance PE2 editing, suggesting that MLH1 variants can inhibit MMR and improve prime editing by forming catalytically impaired MutLα complexes with PMS2 or by saturating the binding of MSH2. Given their domain architecture, MLH1dn and MLH1^NTD^–NLS both inhibit MMR through MSH2 binding. As expected, MLH1dn improved prime editing in a dose-dependent manner (Figure S4B) and did not increase editing in MMR-deficient HCT116 cells (Parsons et al., 1993) (Figure S4C).

Among 55 total dominant negative MMR protein candidates, including additional MLH1 variants and truncations, MLH1dn expressed in *trans* with PE2 provided the greatest average enhancement in PE2 editing efficiency in HEK293T cells (3.2-fold; Figure 4E). We also observed strong improvement of PE2 editing from MLH1^NTD^–NLS expressed in *trans* (2.7-fold on average) and a PE2–P2A–MLH1dn construct (2.4-fold on average). These three constructs also increased PE3 editing efficiency by 1.2-fold on average and reduced indel byproducts by 1.4- to 4.0-fold (Figure 4E). We thus designated PE2 editing with MLH1dn co-expression as the PE4 system, and PE3 editing with MLH1dn co-expression as the PE5 system (Figure 4F). We note that one advantage of MLH1^NTD^–NLS is that it can also enhance prime editing efficiencies with a smaller protein (355 aa) compared to MLH1dn (753 aa). Intriguingly, *MLH1* knockout enhanced PE2 and PE3 editing to a larger degree than MLH1dn co-expression in clonal HeLa cells (Figure S4D), suggesting opportunities for additional prime editing enhancement through further modulation of this pathway.

We further assessed the generality of PE4 and PE5 systems across eight additional single-base substitution edits at different genomic loci in HEK293T cells. On average, PE4 improved editing efficiency over PE2 by 2.0-fold with minimal indels (<0.4% on average; Figure 4G) and was particularly effective at a locus in which complementary-strand nicks yield unproductive editing outcomes (Figure S4E). PE5 improved editing over PE3 by 1.2-fold and enhanced edit/indel purity by 3.0-fold (Figure 4G). MLH1dn also increased efficiency for PE3b (PE5b = PE3b + MLH1dn; Figure S4F), a prime editing strategy that uses a complementary-strand nick specific for the edited sequence to minimize coincident nicks on both strands that promote indel formation (Anzalone et al., 2019). Finally, both human- and mouse-derived MLH1dn improved prime editing efficiency in human HEK293T cells and mouse N2A cells (Figures S4G and S4H). Collectively, these data establish PE4 and PE5 systems that substantially enhance prime editing efficiency and outcome purity at a variety of endogenous genomic loci in mammalian cells.

### Characterization of the types of prime edits enhanced by PE4 and PE5

**Figure 5:**
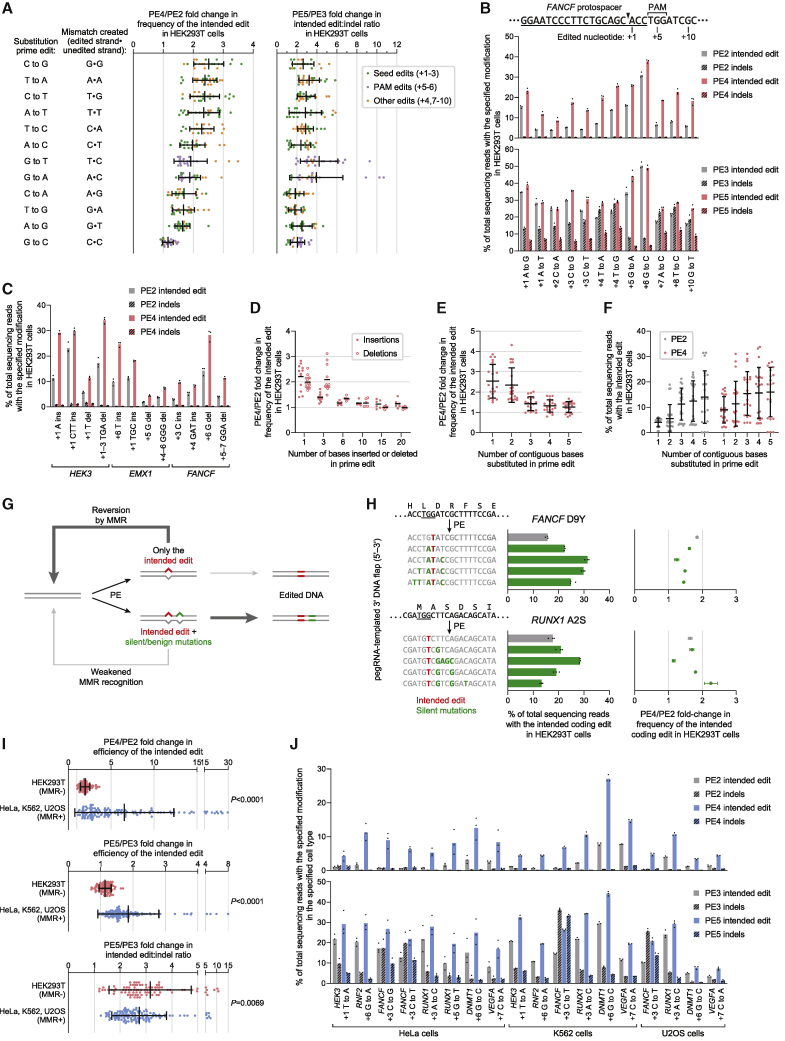
Characterization of PE4 and PE5 across diverse prime edit classes and cell types (A) Summary of prime editing enhancement by PE4 and PE5 compared to PE2 and PE3 for 84 single-base substitution edits (7 for each substitution type) across 7 endogenous sites in HEK293T cells. The grand mean ± SD of all individual values of n = 3 independent replicates are shown. (B) Substitution edits with PE2, PE3, PE4, and PE5 at the *FANCF* locus in HEK293T cells. The black triangle marks the location of the pegRNA-programmed nick. Bars represent the mean of n = 3 independent replicates. (C) PE4 improves 1- and 3-bp indel prime edits compared to PE2 in HEK293T cells (mean of n = 3 independent replicates). (D) PE4 editing enhancement over PE2 across 33 indel prime edits. Lines represent the mean of all individual values of n = 3 independent replicates. (E and F) Summary of PE2 and PE4 editing efficiencies for 35 different substitutions of 1–5 contiguous bases at five endogenous sites in HEK293T cells. Seven pegRNAs were tested for each number of contiguous bases altered. The mean ± SD of all individual values of n = 3 replicates are shown. (G and H) Installation of additional silent or benign mutations near the intended edit can increase editing efficiency by generating a heteroduplex substrate that evades MMR. The PAM sequence (NGG) for each target is underlined. The amino acid sequence of the targeted gene is centered above each triplet DNA codon. Values represent the mean ± SD of n = 3 independent replicates. (I) Summary of PE4 and PE5 editing enhancement in MMR-deficient (MMR−) and MMR-proficient (MMR+) cells. A common set of 30 pegRNAs encoding point mutations were tested in HEK293T and HeLa cells. K562 and U2OS cells were edited with 10 total pegRNAs that are a subset of these 30 pegRNAs. The mean ± SD of all individual values of sets of n = 3 independent replicates are shown. p values were calculated with the Mann-Whitney *U* test. (J) Prime editing with PE2, PE3, PE4, and PE5 in HeLa, K562, and U2OS cells. Bars represent the mean of n = 3 independent replicates.

**Figure S5:**
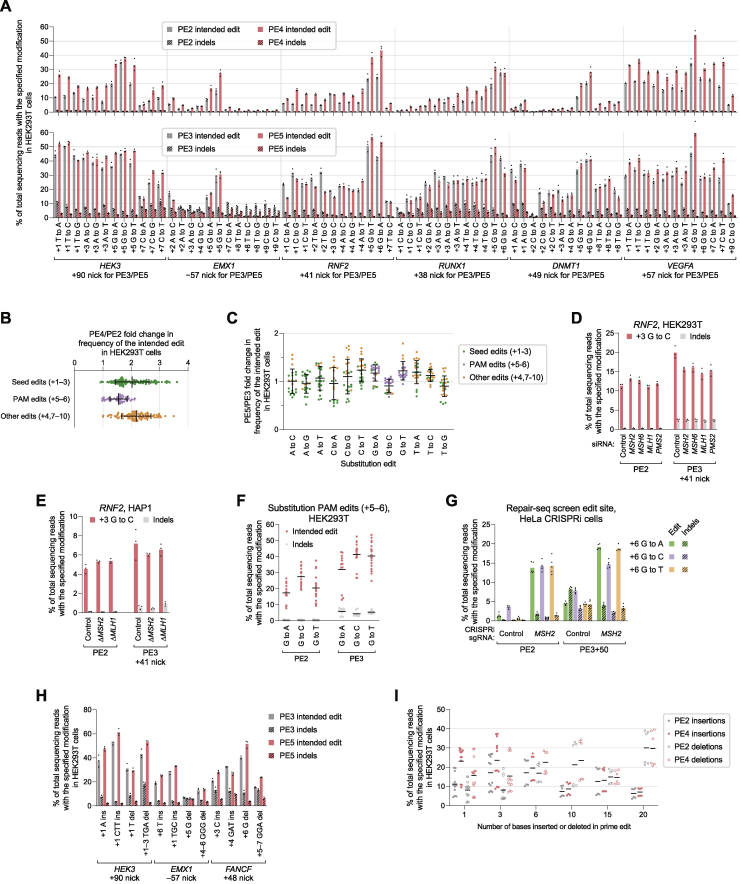

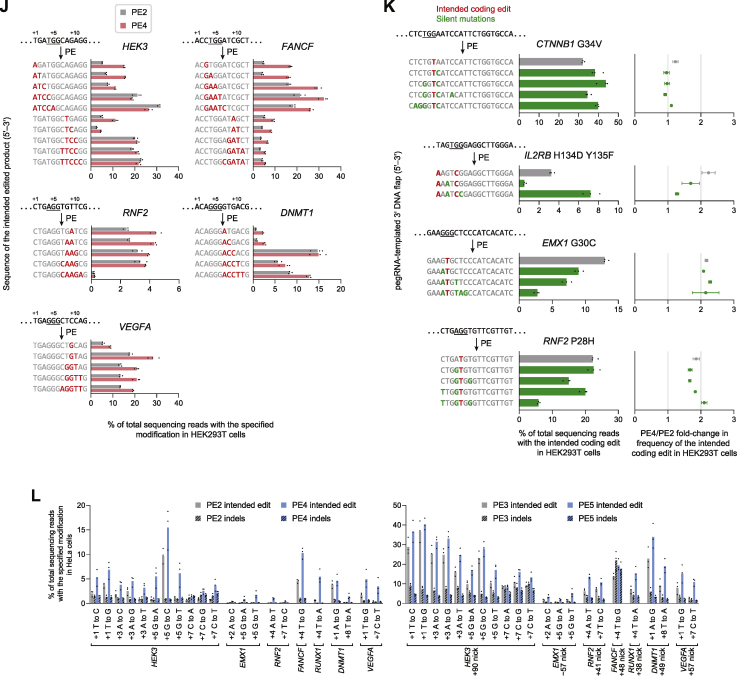
Characterization of PE4 and PE5 systems and improved prime editing efficiency with additional silent mutations, related to Figure 5 (A) Comparison of PE2, PE3, PE4, and PE5 for 84 single-base substitution prime edits across seven endogenous sites in HEK293T cells, supplementing information in Figures 5A, 6D–F, S6B, and S6D. Bars represent the mean of n = 3 independent replicates. (B) Summary of PE4 enhancement in editing efficiency over PE2 for 84 single-base substitution edits across seven endogenous sites in HEK293T cells. PE4/PE2 fold improvements may be lower for PAM edits due to the high basal editing efficiency for PAM edits or the high representation of G⋅C-to-C⋅G edits (five out of 15 in this category). Data represent the mean ± SD of n = 3 independent replicates. (C) Summary of PE5 enhancement in editing efficiency over PE3 for 84 single-base substitution edits in HEK293T cells. The grand mean ± SD of all individual values of n = 3 independent replicates are shown. (D) Effect of siRNA knockdown of MMR genes on G⋅C-to-C⋅G editing at the *RNF2* locus in HEK293T cells. Bars represent the mean of n = 3 independent replicates. (E) Effect of MMR gene knockout on G⋅C-to-C⋅G editing at the *RNF2* locus in HAP1 cells. Δ, gene knockout. Bars represent the mean of n = 3 independent replicates. (F) Efficiencies of single-base substitution prime edits that alter the PAM (+5 G or +6 G bases) of prime editing target protospacers in HEK293T cells. Four G⋅C-to-A⋅T, five G⋅C-to-C⋅G, and six G⋅C-to-T⋅A PAM edits across a combined seven endogenous sites are shown. The mean of all individual values of n = 3 independent replicates are shown. (G) Prime editing at the pre-validated Repair-seq screen edit site with CRISPRi knockdown in HeLa CRISPRi cells. PE2 indicates editing with SaPE2 protein and Sa-pegRNA. PE3+50 indicates editing with SaPE2 protein, Sa-pegRNA, and Sa-sgRNA that programs a +50 complementary-strand nick. Bars represent the mean of n = 5 independent replicates. (H) PE5 improves editing efficiency and reduces indel byproducts compared to PE3 across small insertion and deletion prime edits in HEK293T cells. (I) PE2 and PE4 editing efficiencies at 33 different insertion and deletion prime edits across a combined three endogenous loci. Lines represent the mean of all individual values of n = 3 independent replicates. Data supplements information in Figure 5D. (J) Substitutions of contiguous bases with PE2 and PE4 in HEK293T cells. The top sequence indicates the original, unedited genomic sequence. Numbers denote the position of the edited nucleotide relative to the pegRNA-directed nick site. Nucleotides within the SpCas9 PAM sequence (NGG) are underlined. Sequences of the intended edited product are shown below, with edited nucleotides marked in red. Bars represent the mean of n = 3 independent replicates. Data supplements information in Figures 5E and 5F. (K) Installation of additional silent mutations can increase prime editing efficiency by evading MMR. PE4/PE2 fold-change in editing frequency reflects the extent to which MMR activity impedes the indicated prime edit. Edited nucleotides that make the indicated coding mutation are marked in red, and edited nucleotides that make silent mutations are marked in green. Data represent the mean ± SD of n = 3 independent replicates. (L) Installation of 22 single-base substitution prime edits across seven endogenous sites in HeLa cells with PE2, PE3, PE4, and PE5. Bars represent the mean of n = 3 independent replicates. Data supplements information in Figure 5I.

Next, we studied the extent to which MLH1dn improves prime editing across a wide range of edit types. Since MMR repairs different DNA mismatches with varying efficiencies (Lujan et al., 2014), we anticipated that MLH1dn would more strongly enhance prime edits that proceed through mismatches that are more efficient substrates for MMR. Across 84 pegRNAs that together introduce all 12 possible single-base substitutions at seven endogenous loci in HEK293T cells, PE4 improved editing efficiency by 2.0-fold and reduced indel frequencies from 0.40% to 0.31% compared to PE2 (Figures 5A, 5B, S5A, and S5B). In contrast, PE5 yielded an average 1.2-fold increase in editing and 2.8-fold increase in edit/indel purity relative to PE3 (Figure S5C).

Among the 12 types of base substitutions, G⋅C-to-C⋅G edits, which form C⋅C mismatches after 3′ flap hybridization, were by far the least improved with MLH1dn (1.2-fold comparing PE4 with PE2; Figure 5A), consistent with previous studies establishing that C⋅C mismatches are not efficiently repaired by MMR (Lahue et al., 1989; Su et al., 1988; Thomas et al., 1991). In support of this observation, MMR knockdown in HEK293T cells (Figure S5D) and MMR knockout in HAP1 cells (Figure S5E) did not change the efficiency of a G⋅C-to-C⋅G prime edit at the *RNF2* locus. These findings suggest that G⋅C-to-C⋅G edits more effectively evade MMR and may therefore yield higher basal editing efficiency. Consistent with this possibility, across seven loci, G⋅C-to-C⋅G edits with PE2 were substantially more efficient (27%) than G⋅C-to-A⋅T (18%) or G⋅C-to-T⋅A (20%) edits among prime edits that alter the protospacer adjacent motif (PAM) (Figure S5F). We also compared G⋅C-to-A⋅T, G⋅C-to-C⋅G, and G⋅C-to-T⋅A edits with SaPE2 at the pre-validated screening site in HeLa CRISPRi cells (Figure S1C). PE2 and PE3+50 more efficiently installed the G⋅C-to-C⋅G edit than the G⋅C-to-A⋅T or G⋅C-to-T⋅A edits, consistent with weaker MMR activity at C⋅C mismatches (Figure S5G). Furthermore, CRISPRi knockdown of *MSH2* improved G⋅C-to-A⋅T and G⋅C-to-T⋅A editing efficiencies (16-fold for PE2 and 4.3-fold for PE3+50) to a greater extent than for G⋅C-to-C⋅G (4.0-fold for PE2 and 1.9-fold for PE3+50). Collectively, these data suggest that G⋅C-to-C⋅G prime edits are less susceptible to repair by MMR and are thus installed with higher efficiency.

To determine if MLH1dn could also improve indel prime edits, we installed 1- and 3-bp indels with PE4 and PE5 in HEK293T cells. Across 12 pegRNAs at three loci, PE4 increased average editing efficiency by 2.2-fold over PE2, with no increase in unintended indel frequency, while PE5 increased editing efficiency by 1.2-fold and edit/indel purity by 2.9-fold over PE3 (Figures 5C and S5H). We also tested PE2 and PE4 with 33 pegRNAs that together program 1-, 3-, 6-, 10-, 15-, and 20-bp indels at the *HEK3* and *FANCF* loci. MLH1dn enhancement of prime editing efficiency declined as the length of the indels increased (Figures 5D and S5I), consistent with previous reports that MMR repairs IDLs up to 13 nt in length (Acharya et al., 1996; Genschel et al., 1998; Umar et al., 1994). These results together demonstrate that PE4 and PE5 strategies can enhance small (<15 bp) targeted indels and suggest that longer indel edits benefit less from MLH1dn because their intermediates natively evade MMR.

### Installing additional silent mutations can increase prime editing efficiency by evading MMR

Next, we explored whether other classes of prime edits could bypass MMR. MutSα and MutSβ each recognize specific DNA heteroduplex structures (Gupta et al., 2011; Warren et al., 2007), suggesting that a DNA bubble of contiguous mismatches could weaken recognition by these MMR components. To assess this possibility, we tested PE2 and PE4 with 35 different edits that generate 1- to 5-base contiguous substitutions at five genomic loci in HEK293T cells. Across 2-base substitutions, PE4 yielded 2.3-fold higher editing efficiency than PE2, similar to the 2.4-fold enhancement for single-base substitutions at the same target nucleotides (Figures 5E and S5J). In contrast, PE4 improved the editing of longer 3- to 5-base contiguous substitutions by 1.2- to 1.5-fold relative to PE2. The reduced impact of MMR on these larger edits was also reflected in higher average PE2 editing efficiency for 3- to 5-base contiguous substitutions (13% across 21 edits) compared to 1- or 2-base contiguous substitutions (4.8% across 14 edits) (Figures 5F and S5J).

Next, we asked whether installing additional silent mutations nearby the intended edits could similarly increase prime editing efficiency by weakening repair of the resulting heteroduplex (Figure 5G), even in the absence of MMR inhibition. To test this idea, we designed pegRNAs that program a coding mutation and, optionally, additional silent mutations close to the coding edit (most fewer than 5 bp away). At four of six gene targets, adding these silent mutations increased PE2 efficiency of the desired coding change by an average 1.8-fold for the best pegRNAs at each site (Figures 5H and S5K). Inhibiting MMR with MLH1dn (PE4) improved editing efficiency with the best silent mutations to a lesser extent (1.2-fold on average) compared to only the coding edits (1.7-fold), suggesting that these additional silent mutations enhance editing by evading MMR. Consistent with this mechanism, at the two sites in which silent mutations do not improve editing, the tested silent mutations do not affect MLH1dn enhancement of editing (Figure S5K). Collectively, these findings support that MMR less efficiently repairs heteroduplexes containing three or more contiguous mismatched bases and reveal that the strategic installation of additional benign mutations nearby the desired edit can increase prime editing efficiency by evading MMR, even without manipulating MMR activity.

### PE4 and PE5 strongly improve prime editing in MMR-proficient cell types

HEK293T cells are partially MMR deficient due to hypermethylation of the *MLH1* promoter (Trojan et al., 2002), which may explain higher prime editing efficiency observed in HEK293T cells compared to other mammalian cell types (Anzalone et al., 2019). To evaluate whether MLH1dn improves prime editing to a greater degree in cells without MMR deficiency, we compared prime editing in partially MMR-deficient (MMR−) HEK293T cells and in three MMR-proficient (MMR+) cell types: HeLa (Holmes et al., 1990; Thomas et al., 1991), K562 (Matheson and Hall, 2003), and U2OS (Peng et al., 2014). PE4 enhanced average editing efficiency over PE2 to a much greater extent in MMR+ cells (6.5-fold across 40 edits) than in MMR− cells (2.0-fold across 30 edits) while maintaining minimal indel frequencies (0.61% on average in MMR+ cells; Figures 5I, 5J, and S5L). Similarly, PE5 improved average editing efficiency over PE3 by 1.9-fold in MMR+ cells but only by 1.1-fold in MMR− cells. MLH1dn also increased edit/indel ratios to a similar degree in MMR+ and MMR− cells (2.8-fold and 3.2-fold, respectively; Figure 5I). Intriguingly, although PE4 only increased G⋅C-to-C⋅G editing at *DNMT1* by 1.4-fold over PE2 in HEK293T cells (Figure S5A), we observed a larger improvement in MMR+ cells (averaging 2.7-fold; Figure 5J), suggesting that PE4 and PE5 can enhance prime editing in MMR+ cell types for even classes of edits that evade MMR activity more effectively. Together, this comparison between 70 edits across seven endogenous sites in HEK293T, HeLa, K562, and U2OS cells illustrates that MLH1dn substantially improves prime editing efficiency, especially in MMR-proficient cells, which we expect to include most cell targets of prime editing.

### Effect of MLH1dn on prime editing outcome purity

**Figure 6:**
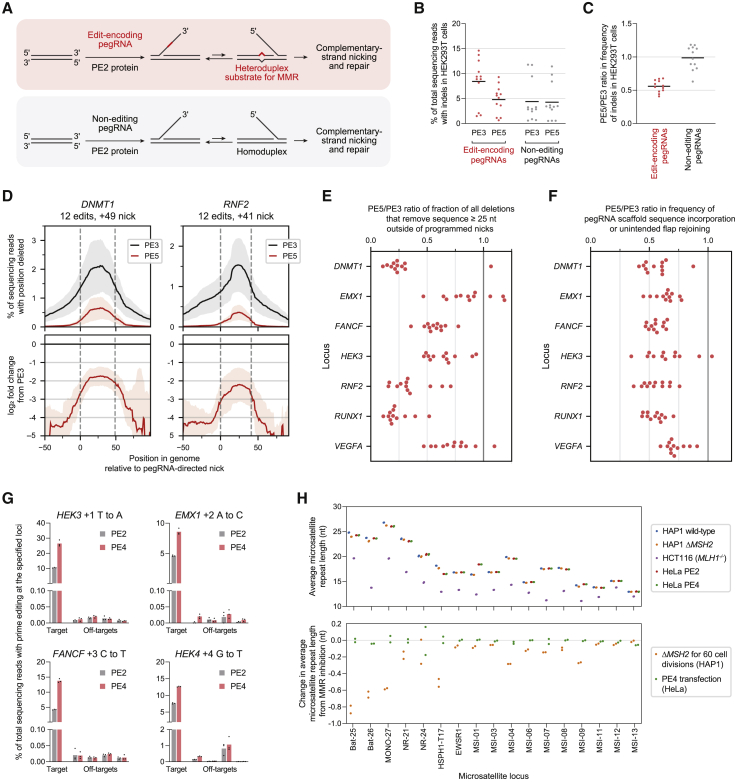
Effect of MLH1dn on prime editing outcome purity and off-targeting (A) Edit-encoding pegRNAs generate a heteroduplex following flap interconversion. Non-editing pegRNAs template a 3′ DNA flap with perfect complementarity to the genomic target site. (B and C) Frequency of indels (B) and ratio of indel frequency (C) from PE3 or PE5 with four edit-encoding pegRNAs that program single-base mutations or four non-editing pegRNAs. Lines indicate mean of all individual values of sets of n = 3 independent replicates. (D) Distribution of deletions at genomic target DNA formed by PE3 and PE5 using 12 substitution-encoding pegRNAs for each locus in HEK293T cells. Dotted lines indicate position of pegRNA- and sgRNA-directed nicks. Data represent the mean ± SD of n = 3 independent replicates. (E and F) PE5/PE3 ratio of frequency of deletions that remove sequence greater than 25 nt outside of pegRNA- and sgRNA-directed nicks (E), and PE5/PE3 ratio of frequency of editing outcomes with unintended pegRNA scaffold sequence incorporation or unintended flap rejoining (F) in HEK293T cells. Each dot represents one of 84 total pegRNAs that program substitution edits (mean of n = 3 independent replicates). (G) Off-target prime editing from PE2 and PE4 in HEK293T cells. Bars represent the mean of n = 3 independent replicates. (H) High-throughput sequencing analysis of microsatellite repeat loci used for clinical diagnosis of MMR deficiency. HAP1 and HeLa cells are MMR proficient, and HCT116 cells have impaired MMR. HAP1 *ΔMSH2* cells underwent 60 cell divisions following *MSH2* knockout. HeLa cells were transiently transfected with PE2 or PE4 components. All values from n = 2 independent replicates are shown.

**Figure S6:**
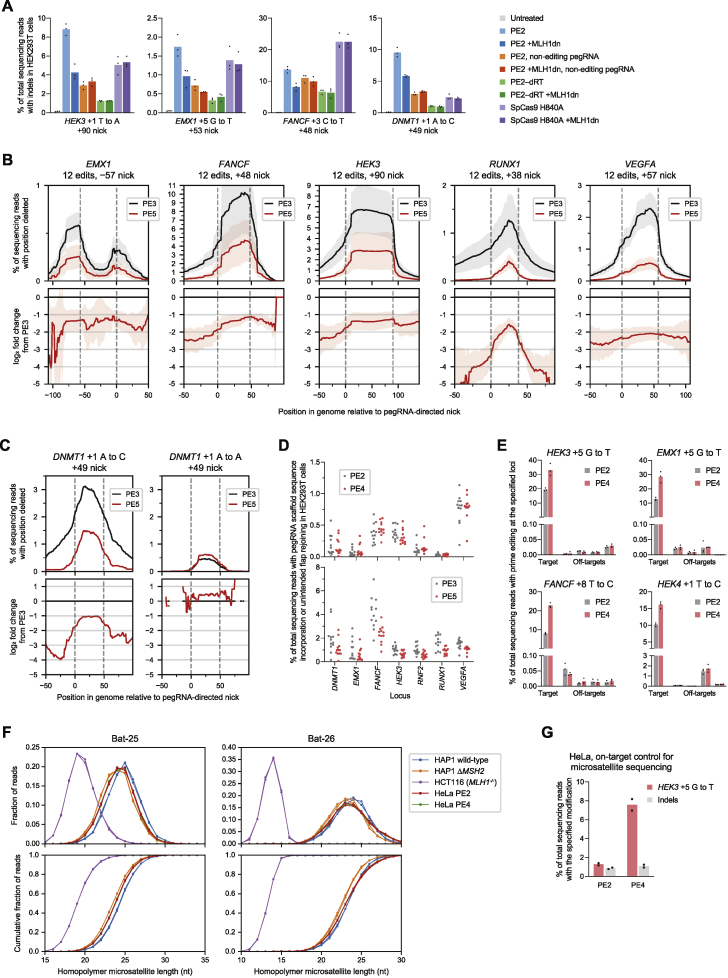
Effect of dominant negative MLH1 on prime editing outcome purity and off-targeting, related to Figure 6 (A) Frequency of indels in HEK293T cells treated with pegRNAs, nicking sgRNAs, and PE2 enzyme, RT-impaired PE2 (PE2–dRT), or nickase Cas9 (SpCas9 H840A), with and without MLH1dn. Non-editing pegRNAs encode a 3′ DNA flap with perfect homology to the genomic target. Bars represent the mean of n = 3 independent replicates. Data supplements information in Figures 6B and 6C. (B) Frequency of deletion as a function of genomic position relative to programmed nicks from PE3 and PE5 in HEK293T cells. 12 different pegRNAs that program single-base substitutions were tested at each indicated endogenous locus. Dotted lines indicate position of pegRNA- and sgRNA-directed nicks. Data represent the mean ± SD of n = 3 independent replicates. (C) Distribution of deletion outcomes from PE3 and PE5 with an edit-encoding and non-editing pegRNA in HEK293T cells. The non-editing pegRNA templates a 3′ DNA flap with perfect complementary to the genomic target sequence. Data represent the mean ± SD of n = 3 independent replicates. (D) Frequency of all prime editing outcomes with unintended pegRNA scaffold sequence incorporation or unintended flap rejoining in HEK293T cells. 12 pegRNAs each programming a different single-base substitution were tested at each of the seven indicated loci. Each dot represents an individual pegRNA at the indicated locus (mean of n = 3 independent replicates). (E) Off-target prime editing by PE2 and PE4 in HEK293T cells. Bars represent the mean of n = 3 independent replicates. (F) Distribution and cumulative distribution of microsatellite repeat lengths in the indicated cell types and treatments. HAP1 and HeLa cells are MMR-proficient, and HCT116 cells have impaired MMR. HAP1 *ΔMSH2* cells underwent 60 cell divisions following knockout of *MSH2*. HeLa cells were transiently transfected with PE2 or PE4 components and grown for 3 days before sequencing. wt, wild-type. All values from n = 2 independent replicates are shown. (G) Prime editing at the on-target locus in HeLa cells transfected with PE2 or PE4 components. Bars represent the mean of n = 2 independent replicates. Microsatellite lengths were assayed from genomic DNA taken from these PE2 and PE4-treated HeLa cells.

Next, we examined in depth how MLH1dn reduces unintended prime editing outcomes. To decouple steps that lead to prime editing from those that lead to indel byproducts, we designed non-editing pegRNAs that template a 3′ DNA flap with perfect complementarity to the target locus and would result in no sequence change at the target locus (Figure 6A). Across four endogenous sites in HEK293T cells, prime editing with non-editing pegRNAs yielded similar indel frequencies for PE3 (4.4%) and PE5 (4.3%; Figures 6B, 6C, and S6A), indicating that MLH1dn does not affect prime editing indel byproducts in the absence of a heteroduplex. In contrast, pegRNAs that program point mutations at these sites induced higher average indels (8.5% with PE3), which were reduced in frequency with MLH1dn (4.8% with PE5). MLH1dn also did not affect indels from PE2 with an inactivated RT (PE2–dRT) or SpCas9 H840A nickase (nCas9; Figure S6A), suggesting that MMR does not repair a doubly nicked intermediate lacking a 3′ flap. These results demonstrate that MMR engagement of the prime editing heteroduplex intermediate stimulates indel products that can be mitigated with PE5.

We also measured the effect of MLH1dn on unintended prime editing outcome classes from 84 pegRNAs encoding single-base substitutions at endogenous loci in HEK293T cells. Similar to results from MMR gene knockdown (Figure 2I), MLH1dn in the PE5 system reduced deletions outside of the pegRNA- and sgRNA-programmed nicks to a greater extent than deletions between these nicks (Figures 6D, 6E, and S6B), but not for a non-editing pegRNA that does not create a mismatch (Figure S6C). In addition, PE5 reduced the average frequency of pegRNA scaffold sequence incorporation (Figure 2C) and unintended flap rejoining outcome categories (Figure 2D) by 1.6-fold compared to PE3 (from 1.8% to 1.0%; Figures 6F and S6D). These outcomes were much rarer in the absence of a complementary-strand nick (0.27% frequency for PE2 and 0.28% for PE4; Figure S6D). Altogether, these data show that PE5 broadly narrows the size of unintended deletions, consistent with our model of DSB intermediate formation during PE3 (Figure S3D), and reduces the frequency of pegRNA scaffold sequence incorporation and unintended flap rejoining compared to PE3.

### Effect of MLH1dn on off-target genomic DNA changes

We next assessed whether MMR component manipulation could influence off-target editing. We tested PE2 and PE4 in HEK293T cells with eight pegRNAs and measured the resulting genomic changes at the four most common Cas9 off-target sites for each targeted locus (Tsai et al., 2017). The average frequency of off-target prime editing remained very low with or without MLH1dn (0.094% with PE2, 0.12% with PE4), while the average efficiency of on-target editing increased from 9.7% for PE2 to 20% for PE4 (Figures 6G and S6E). These data are consistent with previous reports noting the high DNA specificity of prime editing (Anzalone et al., 2019; Jin et al., 2021; Kim et al., 2020) and suggest that MLH1dn does not substantially increase guide-dependent off-target prime editing.

Next, we explored the idea that transient inhibition of MMR with MLH1dn might induce genomic mutations independent of prime editor activity. Because mutations that alter the length of repetitive microsatellite sequences are repaired almost exclusively by MMR (Strand et al., 1993; Tran et al., 1997), microsatellite instability is used clinically as a measure of MMR activity in colorectal cancers (Bacher et al., 2004; Umar et al., 2004). We evaluated microsatellite instability in HAP1, HeLa, and MMR-deficient HCT116 cells by high-throughput sequencing of 17 microsatellites previously validated as biomarkers of MMR activity in tumor specimens (Hempelmann et al., 2015). As expected from their MMR deficiency, HCT116 cells exhibited substantially shorter microsatellite lengths on average (13.9 nt) than HAP1 or HeLa cells (18.4 nt; Figures 6H and S6F). To gauge the sensitivity of this assay, we compared microsatellite instability in wild-type HAP1 cells and monoclonal HAP1 cells grown for 2 months (∼60 cell divisions) following MMR knockout. These MMR knockout cells exhibited a 0.24-nt average decrease in microsatellite length (Figures 6H and S6F), establishing that even recent MMR impairment can be detected through the accumulation of microsatellite length erosion. To assess the effect of transient MLH1dn expression as used in PE4 and PE5 systems, we next measured microsatellite instability in MMR-proficient HeLa cells 3 days after transfection with plasmids encoding PE2 or PE4. Although MLH1dn improved prime editing efficiency from 1.3% (PE2) to 7.6% (PE4) at the on-target locus (Figure S6G), average microsatellite lengths were indistinguishable between PE2- and PE4-treated cells (<0.01 nt of difference; Figures 6H and S6F). These data indicate that transient MLH1dn expression can enhance prime editing without causing detected instability at 17 biomarker microsatellites sensitive to MMR deficiency.

### PEmax systems with optimized editor architecture and synergy with engineered pegRNAs

**Figure S7:**
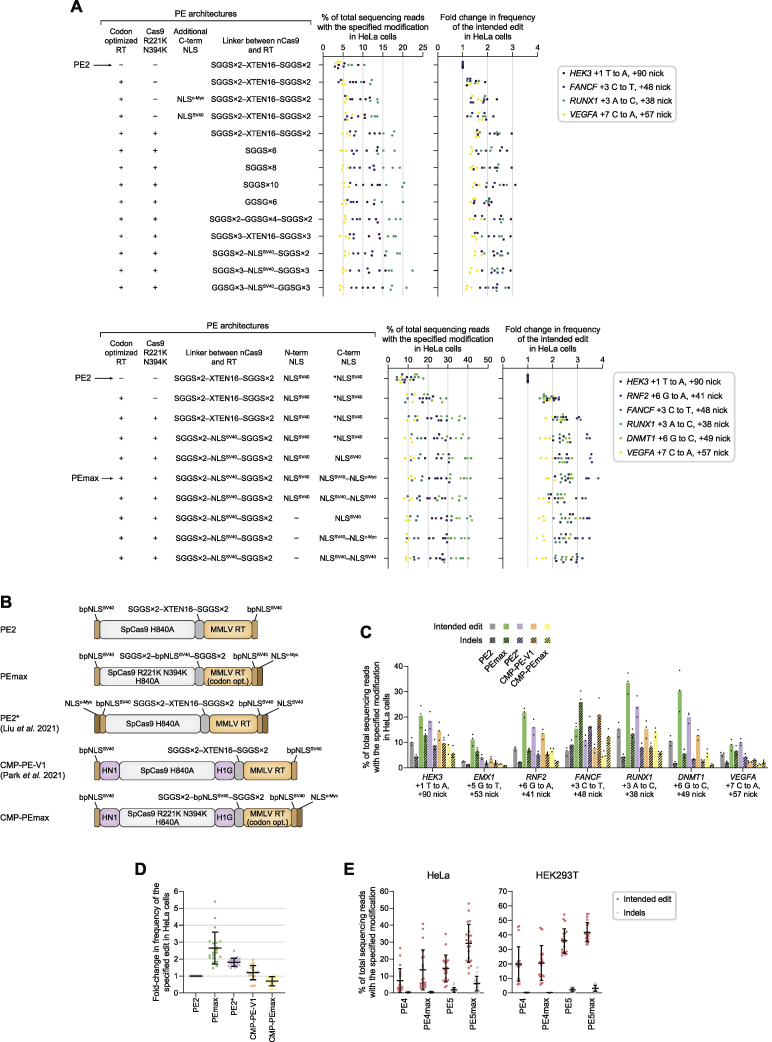

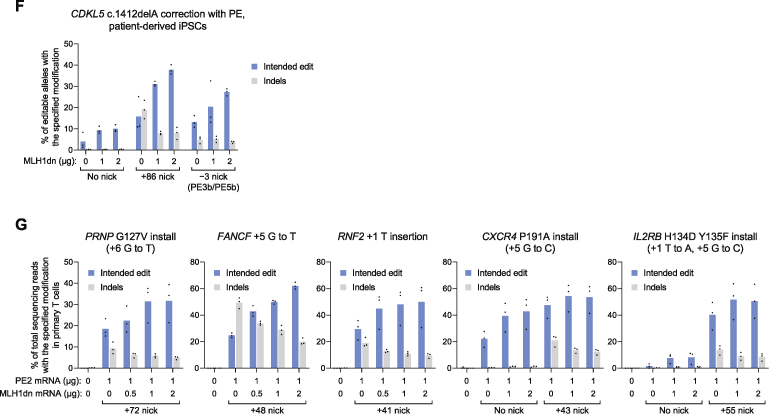
Development of PEmax and application of PE4 and PE5 to primary cell types, related to Figure 7 (A) Screen of prime editor variants for improved editing efficiency with the PE3 system in HeLa cells. All prime editor architectures carry a SpCas9 H840A mutation to prevent nicking of the complementary DNA strand at the target protospacer. NLS^SV40^ indicates the bipartite SV40 NLS. ^∗^NLS^SV40^ contains a 1-aa deletion outside the PKKKRKV NLS^SV40^ consensus sequence. All individual values of n = 3 independent replicates are shown. (B) Architectures of the original PE2 editor (Anzalone et al., 2019), PE2^∗^ (Liu et al., 2021), CMP–PE–V1 (Park et al., 2021), and prime editor variants developed in this work (PEmax, CMP–PEmax). HN1, HMGN1; H1G, histone H1 central globular domain; codon opt., human codon optimized. (C) PEmax outperforms other prime editor architectures tested with the PE3 system in HeLa cells. Bars represent the mean of n = 3 independent replicates. (D) Fold-change in editing efficiency of prime editor architectures compared to PE2 with the PE3 system in HeLa cells. The mean ± SD of all individual values of n = 3 independent replicates are shown. (E) Intended editing and indel frequencies from PE4, PE4max (PE4 editing system with PEmax architecture), PE5, and PE5max (PE5 editing system with PEmax architecture) in HeLa and HEK293T cells cells. Seven substitution prime edits targeting different endogenous loci were tested for each condition. The mean ± SD of all individual values of n = 3 independent replicates are shown. (F) Correction of *CDKL5* c.1412delA via an A⋅T insertion and a G⋅C-to-A⋅T edit in iPSCs derived from a patient heterozygous for the disease allele. Editing efficiencies indicate the percentage of sequencing reads with c.1412delA correction out of editable alleles that carry the mutation. Indel frequencies reflect all sequencing reads that contain any indels that do not map to the c.1412delA allele or wild-type sequence. 1 μg of PE2 mRNA was used in all conditions shown. Bars represent the mean of n = 3 independent replicates. Data supplements information in Figure 7E. (G) Prime editing in primary T cells, supplementing information in Figure 7F. Bars represent the mean of n = 3 independent replicates from different T cell donors.

**Figure 7:**
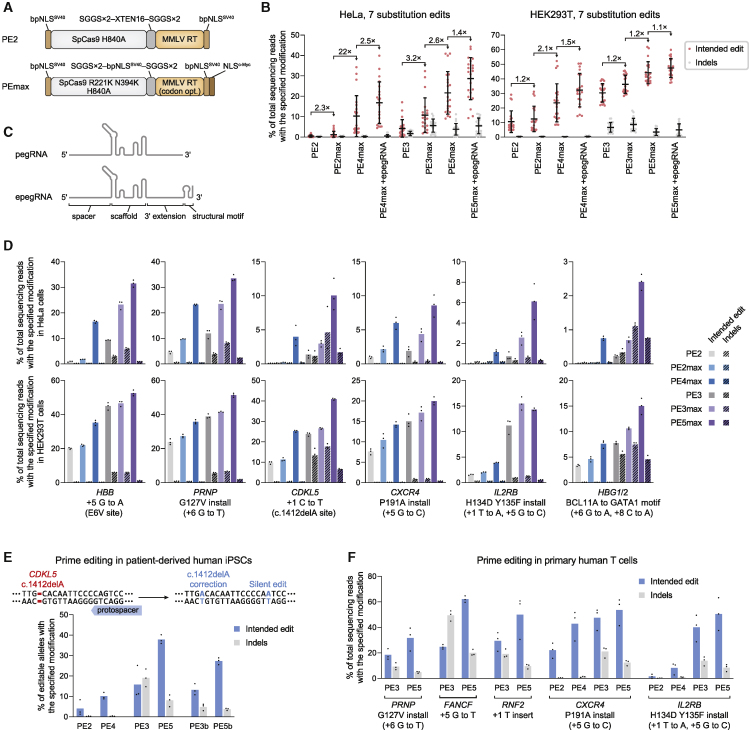
PE4 and PE5 systems and PEmax architecture enhances editing at disease-relevant gene targets and cell types (A) Schematic of PE2 and PEmax editor architectures. bpNLS^SV40^, bipartite SV40 NLS. MMLV RT, Moloney murine leukemia virus RT pentamutant; codon opt., human codon-optimized. (B) Prime editing with PE4 and PE5, PEmax, and epegRNAs at seven endogenous sites in HeLa and HEK293T cells. Fold changes indicate the average of fold increases from each edit tested. The mean ± SD of all individual values of n = 3 independent replicates are shown. (C) Engineered pegRNAs (epegRNAs) contain a 3′ RNA structural motif and improve prime editing performance. (D) Prime editing at therapeutically relevant sites (additional details in STAR Methods) in wild-type HeLa and HEK293T cells. Bars represent the mean of n = 3 independent replicates. (E) Correction of *CDKL5* c.1412delA in iPSCs derived from a patient heterozygous for the allele. Editing efficiencies indicate the percentage of sequencing reads with c.1412delA correction out of editable alleles that carry the mutation. Indel frequencies reflect all sequencing reads that contain any indels. Bars represent the mean of n = 3 independent replicates. (F) Prime editing in primary human T cells. Bars represent the mean of n = 3 different T cell donors.

To further improve prime editing, we optimized the PE2 protein by varying RT codon usage, SpCas9 mutations, NLS sequences, and the length and composition of peptide linkers between nCas9 and RT (Figure S7A). Among 21 such variants tested, we observed the greatest enhancement in editing efficiency from a prime editor architecture that uses a human codon-optimized RT, a 34-aa linker containing a bipartite SV40 NLS (Wu et al., 2009), an additional C-terminal c-Myc NLS (Dang and Lee, 1988), and R221K N394K mutations in SpCas9 previously shown to improve Cas9 nuclease activity (Spencer and Zhang, 2017) (Figures 7A and S7A). At seven target sites tested in HeLa cells, this optimized prime editor architecture (hereafter referred to as PEmax) outperforms other improved prime editor variants, including PE2^∗^, which includes additional NLS sequences (Liu et al., 2021), and CMP–PE–V1, which contains high-mobility peptides (Park et al., 2021) (Figures S7B–S7D). Inserting high-mobility peptides into PEmax (CMP–PEmax) did not further improve prime editing (Figures S7C and S7D).

Across seven substitution edits targeting different loci, using the PEmax architecture with PE2, PE3, PE4, or PE5 systems (hereafter referred to as PE2max, PE3max, PE4max, and PE5max, respectively) increased the average frequency of intended editing by 2.5-fold in HeLa cells and 1.2-fold in HEK293T cells compared the original PE2 editor architecture (Anzalone et al., 2019) (Figures 7B and S7E). PE3max and PE5max also slightly reduced average edit/indel purity by 1.2-fold compared to PE3 and PE5, respectively, which may reflect enhanced nickase activity from the SpCas9 R221K and N394K mutations within the PEmax architecture (Spencer and Zhang, 2017).

We also assessed whether PE4max and PE5max systems can synergize with epegRNAs, which contain an additional 3′ RNA structural motif that increases prime editing efficacy (Nelson et al., 2021) (Figure 7C). Across seven substitution edits, epegRNAs improved PE4max editing efficiency over normal pegRNAs by an average 2.5-fold (HeLa) and 1.5-fold (HEK293T; Figure 7B). Similarly, epegRNAs enhanced PE5max editing over normal pegRNAs by 1.4-fold (HeLa) and 1.1-fold (HEK293T), without affecting edit/indel purity.

Combining all enhancements to prime editing systems described above (MLH1dn, PEmax, and epegRNAs) dramatically improved prime editing performance. PE4max with epegRNAs enhanced editing efficiency by an average of 72-fold in MMR-proficient HeLa cells and 3.5-fold in MMR-deficient HEK293T cells relative to PE2 with normal pegRNAs (Figure 7B). PE5max with epegRNAs also improved editing efficiency over PE3 with pegRNAs by 12-fold (HeLa) and 1.6-fold (HEK293T) on average and increased outcome purity by 4.6-fold (HeLa) and 3.3-fold (HEK293T). Collectively, these results demonstrate that combining PE4/PE5, PEmax, and epegRNA strategies can greatly enhance prime editing outcomes.

### Prime editing of disease-relevant loci and cell types with PE4 and PE5

To establish the applicability of these improved editing systems, we used PE4max and PE5max to edit six genomic sites associated with sickle cell anemia (Ingram, 1956), prion disease (Asante et al., 2015), CDKL5 deficiency disorder (Olson et al., 2019), HIV infection (Liu et al., 2018), and adoptive T cell transfer therapy (Sockolosky et al., 2018). Across these sites, PE4max increased average prime editing efficiency over PE2 by 29-fold in HeLa cells and 2.1-fold in HEK293T cells (Figure 7D). Notably, PE4max editing efficiencies (8.6% editing and 0.19% indels in HeLa, 20% editing and 0.26% indels in HEK293T) were similar to or exceeded those of PE3 (4.5% editing and 1.5% indels in HeLa and 24% editing and 5.4% indels in HEK293T) but with far fewer indels. In addition, PE5max improved disease-relevant allele conversion over PE3 by an average of 6.1-fold (HeLa) and 1.5-fold (HEK293T) and enhanced edit/indel purity by 6.4-fold (HeLa) and 3.5-fold (HEK293T; Figure 7D). Taken together, these results demonstrate that PE4max and PE5max support substantially higher prime editing performance compared to PE2 and PE3 at therapeutically relevant gene targets in cell culture.

Next, we used PE4 and PE5 to correct the pathogenic *CDKL5* c.1412delA mutation in human iPSCs derived from a heterozygous patient (Chen et al., 2021). Electroporation of iPSCs with *in vitro*-transcribed PE2 mRNA and synthetic pegRNAs and nicking sgRNAs (PE3) yielded 17% correction of editable pathogenic alleles and 20% total indel products (Figures 7E and S7F). Co-electroporation of these components with MLH1dn mRNA (PE5) elevated correction efficiency to 34% and lowered the frequency of indels to 6.1%. PE4 and PE5b systems also improved allele correction by 2.5-fold and 2.1-fold over PE2 and PE3b, respectively, with few indels (0.34% from PE4 and 3.8% from PE5b). Thus, across these prime editing systems tested, MLH1dn enhances *CDKL5* c.1412delA correction by 2.2-fold in efficiency and 3.6-fold in outcome purity in patient-derived iPSCs.

Lastly, we tested mRNA delivery of PE5 in primary human T cells to introduce the protective *PRNP* G127V mutation, a G⋅C-to-T⋅A transversion at *FANCF*, and a 1-bp insertion at *RNF2*. We also installed the protective *CXCR4* P191A allele that prevents HIV infection (Liu et al., 2018), and the *IL2RB* H134D Y135F variant that enables orthogonal IL-2 T cell stimulation (Sockolosky et al., 2018) using PE4 and PE5. Across these five sites, we found that MLH1dn in the PE4 or PE5 systems enhanced editing efficiency by 2.2-fold and the edit/indel ratio by 2.7-fold, achieving an average of 46% editing with 11% indels from PE5 (Figures 7F and S7G). Collectively, these results across six loci in human iPSCs and primary T cells establish PE4 and PE5 as enhanced prime editing systems that enable substantially greater editing efficiency and outcome purity in cell types relevant to the study and potential treatment of genetic disease.

## Discussion

Using pooled CRISPRi screens, we discovered that MMR activity strongly suppresses the efficiency and outcome purity of substitution prime edits. These insights informed the development of PE4 and PE5 systems that co-express MLH1dn to transiently inhibit MMR, enhance prime editing efficacy, and reduce indels without inducing substantial off-target genomic changes. Optimization of the prime editor protein resulted in a PEmax architecture that can synergize with PE4 and PE5 systems and with epegRNAs (Nelson et al., 2021) to further enhance prime editing performance. Together, the model for DNA repair of prime editing supported by these findings, the PE4 and PE5 strategies developed to circumvent a prime editing bottleneck, and the improved PEmax prime editor architecture described here substantially advance the utility of prime editing for precision manipulation of the genome.

Broad characterization of PE4 and PE5 across 191 diverse prime edits reveals that prime editors can install certain types of edits with higher efficiency due to the ability of the corresponding prime editing intermediates to evade MMR. In addition to edit type, other properties could also affect the sensitivity of prime editing to MMR, such as the sequence context of the target site. Moreover, MMR more efficiently repairs early replicating euchromatin (Supek and Lehner, 2015) and lagging strand DNA during replication (Lujan et al., 2014). A systematic study across a larger set of edits will be needed to comprehensively elucidate these edit type, sequence context, and locus state dependencies on MMR and prime editing. Repair-seq may also be applied in the future to illuminate other classes of prime edits, such as long insertions or deletions, and suggest additional improved prime editing systems.

Our study of the types of prime editing intermediates that are repaired by MMR allows researchers to design prime editing experiments to evade MMR, even without expression of MLH1dn. We show that strategically installing additional nearby silent mutations can enhance prime editing outcomes by avoiding MMR reversal of prime editing intermediates. Other modalities for MMR inhibition may also prove beneficial for prime editing. Although no small molecules that selectively target MMR have been reported, chemical inhibitors would be useful in applications limited by MLH1dn delivery. For uses such as viral delivery that maintain long-term prime editor expression, RNA interference may offer an alternate means to transiently knock down MMR activity.

PE4 and PE5 systems powerfully enhance prime editing performance and precision in seven mammalian cell types tested and synergize with improvements from PEmax and epegRNAs. PE4max with epegRNAs uniquely enables efficient prime editing with low indel byproducts, particularly in cells with active MMR, making it most suitable for gene editing applications that require high outcome purity or cannot use nicking sgRNAs. In comparison, PE5max with epegRNAs achieves the highest levels of prime editing with reduced indel outcomes compared to PE3 systems. We therefore recommend the use of PE5max and epegRNAs for most prime editing applications.

## Limitations of the study

While complete knockout of MMR activity enhances prime editing, it remains unknown how a heteroduplex prime editing intermediate is resolved in the absence of MMR. Future work in post-mitotic MMR-deficient cells could illuminate the requirement of cellular factors or DNA replication for resolving the heteroduplex intermediate. In addition, we showed that transient MLH1dn expression minimally perturbs microsatellites sensitive to MMR activity, though the potential impact of MMR deficiency on genomic mutation rates is well documented (Lujan et al., 2014; Zou et al., 2021). An analysis of genome-wide mutations induced by transient PE4 and PE5 expression would more sensitively quantify their off-target editing consequences. Lastly, we demonstrated that installing additional mutations near the intended edit can increase prime editing efficiency by evading MMR. To generalize this strategy, a larger set of edits may need to be tested to establish design rules for silent or benign edits that optimally evade MMR.

## STAR★Methods

### Key resources table

**Table:** 

REAGENT or RESOURCE	SOURCE	IDENTIFIER
**Bacterial and virus strains**		

One Shot Mach1 T1 Phage-Resistant Chemically Competent *E. coli*	Thermo Fisher Scientific	Cat#C862003
MegaX DH10B T1 R Electrocomp Cells	Thermo Fisher Scientific	Cat#C640003

**Chemicals, peptides, and recombinant proteins**		

USER enzyme	New England BioLabs	Cat#M5505S
DpnI	New England BioLabs	Cat#R0176S
BsaI-HFv2	New England BioLabs	Cat#R3733S
T4 DNA Ligase	New England BioLabs	Cat#M0202S
BstXI	Thermo Fisher Scientific	Cat#ER1021
Bpu1102I (BlpI)	Thermo Fisher Scientific	Cat#ER0091
Lipofectamine 2000	Thermo Fisher Scientific	Cat#11668019
Lipofectamine 3000	Thermo Fisher Scientific	Cat#L3000015
Lipofectamine RNAiMAX	Thermo Fisher Scientific	Cat#13778150
TransIT-HelaMONSTER	Mirus Bio	Cat#MIR 2904
TrypLE	Thermo Fisher Scientific	Cat#12605010
Polybrene (Hexadimethrine bromide)	Sigma-Aldrich	Cat#107689-10G
Puromycin Dihydrochloride	Thermo Fisher Scientific	Cat#A1113803
Blasticidin S HCl	Thermo Fisher Scientific	Cat#A1113903
Penicillin-Streptomycin	Thermo Fisher Scientific	Cat#15070063
L-Glutamine	Corning	Cat#25-005-Cl
Proteinase K	New England BioLabs	Cat#P8107S
ViralBoost Reagent	ALSTEM	Cat#VB100
SPRIselect	Beckman Coulter	Cat#A63881
AMPure XP	Beckman Coulter	Cat#B23318
CleanCap Reagent AG	TriLink BioTechnologies	Cat#N-7113
N^1^-Methylpseudouridine-5′-Triphosphate	TriLink BioTechnologies	Cat#N-1081
LiCl Precipitation Solution (7.5 M)	Thermo Fisher Scientific	Cat#AM9480
StemFlex medium	Thermo Fisher Scientific	Cat#A3349401
Geltrex Basement Membrane Matrix	Thermo Fisher Scientific	Cat#A1413301
DMEM/F12, GlutaMAX supplement	Thermo Fisher Scientific	Cat#10565018
Gentle Cell Dissociation Reagent	STEMCELL Technologies	Cat#07174
rhLaminin-521	Thermo Fisher Scientific	Cat#A29249
Y-27632	Cayman Chemical	Cat#10005583 (CAS#129830-38-2)
Accutase	Innovative Cell Technologies	Cat#AT104
Lymphoprep density gradient medium	STEMCELL Technologies	Cat#07801
Dynabeads Human T-Expander CD3/CD28	Thermo Fisher Scientific	Cat#11141D
Human AB Serum	Valley Biomedical	Cat#HP1022HI
GlutaMAX supplement	Thermo Fisher Scientific	Cat#35050061
*N*-Acetyl-L-cysteine	Sigma-Aldrich	Cat#A7250-100G
Recombinant Human IL-2	Peprotech	Cat#200-02
Recombinant Human IL-7	Peprotech	Cat#200-07
Recombinant Human IL-15	Peprotech	Cat#200-15
CleanCap EGFP mRNA	TriLink BioTechnologies	Cat#L-7601

**Critical commercial assays**		

Phusion U Multiplex PCR Master Mix	Thermo Fisher Scientific	Cat#F562L
Q5 High-Fidelity 2 × Master Mix	New England BioLabs	Cat#M0492L
Phusion High-Fidelity DNA Polymerase	Thermo Fisher Scientific	Cat#F530S
NEBNext Ultra II Q5 Master Mix	New England BioLabs	Cat#M0544L
QIAquick PCR Purification Kit	QIAGEN	Cat#28104
QIAquick Gel Extraction Kit	QIAGEN	Cat#28704
NucleoSpin Gel and PCR Clean-up	Macherey-Nagel	Cat#74609.50
QIAGEN Plasmid *Plus* Midi Kit	QIAGEN	Cat#12943
QIAGEN Plasmid *Plus* Maxi Kit	QIAGEN	Cat#12963
ZymoPURE II Plasmid Maxiprep Kit	Zymo Research	Cat#D4202
PureYield Plasmid Miniprep System	Promega Corporation	Cat#A1222
Macherey-Nagel NucleoSpin Blood XL, Maxi kit	Machery-Nagel	Cat#740950.50
SE Cell Line 4D-Nucleofector X Kit S	Lonza	Cat#V4XC-1032
SF Cell Line 4D-Nucleofector X Kit S	Lonza	Cat#V4XC-2032
Bioanalyzer High Sensitivity DNA Kit	Agilent Technologies	Cat#5067-4626
SYBR Green Fast Advanced Cells-to-CT Kit	Thermo Fisher Scientific	Cat#A35380
NEB T7 HiScribe Kit	New England BioLabs	Cat#E2040S
EasySep Human T Cell Isolation Kit	STEMCELL Technologies	Cat#17951
NEON Transfection System 10 μL Kit	Thermo Fisher Scientific	Cat#MPK1025
NEON Transfection System	Thermo Fisher Scientific	Cat#MPK5000
PureLink Genomic DNA Mini Kit	Thermo Fisher Scientific	Cat#K182002
MiSeq Reagent Kit v2 (300-cycles)	Illumina	Cat#MS-102-2002
MiSeq Reagent Micro Kit v2 (300-cycles)	Illumina	Cat#MS-103-1002
NovaSeq 6000 S1 Reagent Kit v1.5 (300-cycles)	Illumina	Cat#20028317

**Deposited data**		

Amplicon sequencing data	This paper	PRJNA735408
Data from Repair-seq screens	This paper	PRJNA734952
Processed data from Repair-seq screens	This paper	DOI: https://doi.org/10.5281/zenodo.5551032

**Experimental models: Cell lines**		

Human (female): HEK293T	ATCC	Cat#CRL-3216
Human (female): HeLa expressing dCas9–BFP–KRAB (Addgene #46911)	Gilbert et al., 2013	N/A
Human (female): K562 expressing dCas9–BFP–KRAB (Addgene #46911)	Gilbert et al., 2014	N/A
Human (female): HeLa	ATCC	Cat#CCL-2
Human: HAP1 parental control	Horizon Discovery	Cat#C631
Human: HAP1 *ΔMSH2*	Horizon Discovery	Cat#HZGHC024799c006
Human: HAP1 *ΔMLH1*	Horizon Discovery	Cat#HZGHC000343c021
Human (male): HCT116	ATCC	Cat#CCL-247
Mouse (male): N2A	ATCC	Cat#CCL-131
Human (female): K562	ATCC	Cat#CCL-243
Human (female): U2OS	ATCC	Cat#HTB-96
Human (female): *CDKL5* deficiency disorder patient-derived induced pluripotent stem cell	Coriell Institute	Cat#OR00007

**Oligonucleotides**		

ON-TARGETplus Human MSH2 siRNA	Horizon Discovery	Cat#L-003909-00-0005
ON-TARGETplus Human MSH6 siRNA	Horizon Discovery	Cat#L-019287-00-0005
ON-TARGETplus Human MLH1 siRNA	Horizon Discovery	Cat#L-003906-00-0005
ON-TARGETplus Human PMS2 siRNA	Horizon Discovery	Cat#L-010032-00-0005
ON-TARGETplus Non-targeting Control Pool siRNA	Horizon Discovery	Cat#D-001810-10-05
*CDKL5* c.1412delA correction with silent edit epegRNA: mG^∗^mA^∗^mG^∗^rGrGrArCr UrCrCrUrArGrArGrGrArCrUrGrGrUrUrUr UrArGrArGrCrUrArGrArArArUrArGrCrArAr GrUrUrArArArArUrArArGrGrCrUrArGrUrCr CrGrUrUrArUrCrArArCrUrUrGrArArArArAr GrUrGrGrCrArCrCrGrArGrUrCrGrGrUrGrCr ArUrArUrUrGrArCrArCrArArUrUrCrCrCrCr ArArUrCrCrUrCrUrArGrGrArGrUrCrArArAr ArArCrArCrGrUrCrArGrGrGrUrCrArGrGrAr GrCrCrCrCrCrCrCrCrCrUrGrCrArCrCrCrAr GrGrArArArArCrCrCrUrCrArArArGrUrCrGr GrGrGrGrGrCrArA^∗^mC^∗^mC^∗^mC	Integrated DNA Technologies	N/A
*CDKL5* +85 nick sgRNA: mG^∗^mC^∗^mA^∗^rGr ArArCrCrGrCrCrArCrUrCrArUrUrCrArGr UrUrUrUrArGrArGrCrUrArGrArArArUrAr GrCrArArGrUrUrArArArArUrArArGrGrCr UrArGrUrCrCrGrUrUrArUrCrArArCrUr UrGrArArArArArGrUrGrGrCrArCrCrGr ArGrUrCrGrGrUrGrCrUmU^∗^mU^∗^mU	Synthego Corporation	N/A
*CDKL5* −3 nick sgRNA (for PE3b/PE5b): mA^∗^mC^∗^mA^∗^rCrArArUrUrCrCrCrCrArArUr CrCrUrCrUrGrUrUrUrUrArGrArGrCrUrAr GrArArArUrArGrCrArArGrUrUrArArArAr UrArArGrGrCrUrArGrUrCrCrGrUrUrAr UrCrArArCrUrUrGrArArArArArGrUrGrGr CrArCrCrGrArGrUrCrGrGrUrGrCrUmU^∗^ mU^∗^mU	Synthego Corporation	N/A
*PRNP* G127V install pegRNA: mG^∗^mC^∗^ mA^∗^rGrUrGrGrUrGrGrGrGrGrGrCrCrUr UrGrGrGrUrUrUrUrArGrArGrCrUrArGr ArArArUrArGrCrArArGrUrUrArArArArUr ArArGrGrCrUrArGrUrCrCrGrUrUrArUrCr ArArCrUrUrGrArArArArArGrUrGrGrCrArCr CrGrArGrUrCrGrGrUrGrCrArUrGrUrArGr ArCrGrCrCrArArGrGrCrCrCrCrCrC^∗^mA^∗^ mC^∗^mC	Integrated DNA Technologies	N/A
*PRNP* +72 nick sgRNA: mG^∗^mC^∗^mA^∗^rUrGr UrUrUrUrCrArCrGrArUrArGrUrArArGrUrUr UrUrArGrArGrCrUrArGrArArArUrArGrCrAr ArGrUrUrArArArArUrArArGrGrCrUrArGrUr CrCrGrUrUrArUrCrArArCrUrUrGrArArArAr ArGrUrGrGrCrArCrCrGrArGrUrCrGrGmUr GrCrUmU^∗^mU^∗^mU	Synthego Corporation	N/A
*FANCF* +5 G to T pegRNA: mG^∗^mG^∗^ mA^∗^rArUrCrCrCrUrUrCrUrGrCrArGrCr ArCrCrGrUrUrUrUrArGrArGrCrUrArGrAr ArArUrArGrCrArArGrUrUrArArArArUrArAr GrGrCrUrArGrUrCrCrGrUrUrArUrCrArAr CrUrUrGrArArArArArGrUrGrGrCrArCrCr GrArGrUrCrGrGrUrGrCrGrGrArArArArGr CrGrArUrCrArArGrGrUrGrCrUrGrCrArGr ArArG^∗^mG^∗^mG^∗^mA	Agilent Technologies	N/A
*FANCF* +48 nick sgRNA: mG^∗^mG^∗^ mG^∗^rGrUrCrCrCrArGrGrUrGrCrUrGr ArCrGrUrGrUrUrUrUrArGrArGrCrUr ArGrArArArUrArGrCrArArGrUrUrArAr ArArUrArArGrGrCrUrArGrUrCrCrGr UrUrArUrCrArArCrUrUrGrArArArAr ArGrUrGrGrCrArCrCrGrArGrUrCrGr GrUrGrCrUmU^∗^mU^∗^mU	Synthego Corporation	N/A
*RNF2* +1 T insertion pegRNA: mG^∗^mU^∗^ mC^∗^rArUrCrUrUrArGrUrCrArUrUrArCr CrUrGrGrUrUrUrUrArGrArGrCrUrArGr ArArArUrArGrCrArArGrUrUrArArArArUr ArArGrGrCrUrArGrUrCrCrGrUrUrArUr CrArArCrUrUrGrArArArArArGrUrGrGr CrArCrCrGrArGrUrCrGrGrUrGrCrArAr CrGrArArCrArCrCrUrCrArGrArGrUrAr ArUrGrArCrUrArArG^∗^mA^∗^mU^∗^mG	Integrated DNA Technologies	N/A
*RNF2* +41 nick sgRNA: mU^∗^mC^∗^mA^∗^ rArCrCrArUrUrArArGrCrArArArArCrAr UrGrUrUrUrUrArGrArGrCrUrArGrArAr ArUrArGrCrArArGrUrUrArArArArUrAr ArGrGrCrUrArGrUrCrCrGrUrUrArUr CrArArCrUrUrGrArArArArArGrUrGr GrCrArCrCrGrArGrUrCrGrGrUrGrCrU mU^∗^mU^∗^mU	Synthego Corporation	N/A
*CXCR4* P191A install pegRNA: mC^∗^ mA^∗^mA^∗^rCrCrArCrCrCrArCrArArGr UrCrArUrUrGrGrUrUrUrUrArGrArGr CrUrArGrArArArUrArGrCrArArGrUr UrArArArArUrArArGrGrCrUrArGrUr CrCrGrUrUrArUrCrArArCrUrUrGrAr ArArArArGrUrGrGrCrArCrCrGrArGr UrCrGrGrUrGrCrUrGrArCrCrGrCrUr UrCrUrArCrGrCrCrArArUrGrArCrUr UrGrUrGrGrGrU^∗^mG^∗^mG^∗^mU	Integrated DNA Technologies	N/A
*CXCR4* +43 nick sgRNA: mC^∗^mA^∗^mU^∗^rCrUr UrUrGrCrCrArArCrGrUrCrArGrUrGrGrUrUr UrUrArGrArGrCrUrArGrArArArUrArGrCrAr ArGrUrUrArArArArUrArArGrGrCrUrArGrUr CrCrGrUrUrArUrCrArArCrUrUrGrArArArAr ArGrUrGrGrCrArCrCrGrArGrUrCrGrGrUrGr CrUmU^∗^mU^∗^mU	Synthego Corporation	N/A
*IL2RB* H134D Y135F install pegRNA: mC^∗^ mC^∗^mA^∗^rGrGrUrGrUrCrUrUrUrCrArArArGr UrArGrGrUrUrUrUrArGrArGrCrUrArGrArAr ArUrArGrCrArArGrUrUrArArArArUrArArGr GrCrUrArGrUrCrCrGrUrUrArUrCrArArCrUr UrGrArArArArArGrUrGrGrCrArCrCrGrArGr UrCrGrGrUrGrCrUrCrCrCrArArGrCrCrUrCr CrGrArCrUrUrCrUrUrUrGrArArArGrA^∗^mC^∗^ mA^∗^mC	Integrated DNA Technologies	N/A
*IL2RB* +55 nick sgRNA: mC^∗^mU^∗^mC^∗^rCr CrUrCrCrArArGrUrUrGrUrCrCrArCrGrGrUr UrUrUrArGrArGrCrUrArGrArArArUrArGrCr ArArGrUrUrArArArArUrArArGrGrCrUrArGr UrCrCrGrUrUrArUrCrArArCrUrUrGrArArAr ArArGrUrGrGrCrArCrCrGrArGrUrCrGrGrUr GrCrUmU^∗^mU^∗^mU	Synthego Corporation	N/A

**Recombinant DNA**		

pCMV–PE2	Anzalone et al., 2019	132775
pMD2.G	Addgene	12259
psPAX	Addgene	12260
pLX_311-Cas9	Addgene	96924
pCMV–SaPE2	Addgene	174817
pCMV–SaPE2–P2A–BSD	Addgene	174818
pCMV–PE2–P2A–BSD	Addgene	174819
pCMV–PEmax	Addgene	174820
pCMV–PEmax–P2A–BSD	Addgene	174821
pEF1α–hMLH1dn (original codon)	Addgene	174823
pEF1α–hMLH1dn (codon opt.)	Addgene	174824
pEF1α–mMLH1dn (codon opt.)	Addgene	174825
pEF1α–hMLH1NTD–NLS (codon opt.)	Addgene	174826
pCMV–PE2–P2A–MLH1dn (codon opt.)	Addgene	174827
pCMV–PEmax–P2A–MLH1dn (codon opt.)	Addgene	174828
pPC1000 (Repair-seq sgRNA–prime edit site)	Addgene	174829

**Software and algorithms**		

CRISPResso2	Clement et al., 2019	https://github.com/pinellolab/CRISPResso2
Prism	GraphPad	https://www.graphpad.com
Repair-seq processing software	(Hussmann et al., 2021)	https://github.com/jeffhussmann/repair-seq (DOI: https://doi.org/10.5281/zenodo.5534778)

### Resource availability

#### Lead contact

Please direct requests for resources and reagents to Lead Contact David R. Liu (drliu@fas.harvard.edu).

#### Materials availability

Plasmids generated in this study are available from Addgene (additional details provided in the Key resources table).

### Experimental model and subject details

#### Culture conditions for immortalized cell lines

HEK293T, HeLa, HCT116, and N2A cells were cultured in Dulbecco’s Modified Eagle Medium (DMEM) plus GlutaMAX (Thermo Fisher Scientific) supplemented with 10% fetal bovine serum (FBS) (Thermo Fisher Scientific). HeLa dCas9–BFP–KRAB cells were cultured in DMEM plus GlutaMAX supplemented with 10% FBS, 100 U mL^-1^ penicillin, and 100 μg mL^-1^ streptomycin (Thermo Fisher Scientific). K562 dCas9–BFP–KRAB and K562 cells were cultured in Roswell Park Memorial Institute (RPMI) 1640 medium (Thermo Fisher Scientific) supplemented with 10% FBS, 100 U mL^-1^ penicillin, 100 μg mL^-1^ streptomycin (Thermo Fisher Scientific), and 292 μg mL^-1^ L-Glutamine (Corning). All HAP1 cell types were cultured in Iscove’s Modified Dulbecco’s Medium (IMDM) plus GlutaMAX (Thermo Fisher Scientific) supplemented with 10% FBS. U2OS cells were cultured in McCoy’s 5A medium (GIBCO) supplemented with 10% FBS, 100 U mL^-1^ penicillin, and 100 μg mL^-1^ streptomycin (Thermo Fisher Scientific). HeLa dCas9–BFP–KRAB and K562 dCas9–BFP–KRAB cell lines were verified by short tandem repeat marker testing. All cell types were passaged every 2–3 days, maintained below 80% confluency, cultured at 37°C with 5% CO_2_, and tested negative for mycoplasma.

#### Isolation of primary human T cells

Peripheral blood mononuclear cells (PBMCs) were isolated from the buffy coat of healthy donors (Memorial Blood Centers in St. Paul, Minnesota) by density centrifugation using Lymphoprep density gradient medium (STEMCELL Technologies) and SepMate tubes (STEMCELL Technologies). T cells were isolated from PBMCs using the EasySep Human T Cell Isolation Kit (STEMCELL Technologies).

#### Culture conditions for human patient-derived induced pluripotent stem cells

All iPSC culturing work was performed by staff at the Human Neuron Core at Boston Children’s Hospital following institutional guidelines and under institutional approvals (IRB#: P00016119). A clonal iPS cell line, MAN0855-01 #A (Coriell Institute #OR00007), was expanded from a female *CDKL5* deficiency disorder patient carrying a heterozygous *CDKL5* c.1412delA p.D471fs mutation on the X chromosome (Chen et al., 2021). MAN0855-01 #A was previously verified to express the mutant *CDKL5* transcript by Sanger sequencing of cDNA. The MAN0855-01 #A iPS cell line was cultured in StemFlex medium (Thermo Fisher Scientific) on Geltrex (Thermo Fisher Scientific) diluted 1:50 in DMEM/F12 (Thermo Fisher Scientific) and coated according to the manufacturer’s protocol. For regular maintenance, iPS cell colonies were clump-passaged using Gentle Cell Dissociation Reagent (STEMCELL Technologies) at 80% confluency every 5–7 days.

### Method details

#### General methods and molecular cloning

Lentiviral transfer plasmids and plasmids for mammalian expression of prime editors and other proteins were cloned using uracil excision (USER) assembly (Cavaleiro et al., 2015). Briefly, DNA fragments were amplified with deoxyuracil-containing primers (Integrated DNA Technologies) using the uracil tolerant Phusion U Green Multiplex PCR Master Mix (Thermo Fisher Scientific). Deoxyuracil-incorporated DNA fragments were assembled with USER enzyme (New England BioLabs) and DpnI (New England BioLabs) according to the manufacturer’s protocol using junctions with a melting temperature of 42–60°C, followed by transformation into cells. All prime editor constructs were cloned into the pCMV–PE2 vector backbone (Anzalone et al., 2019) (Addgene #132775) under constitutive expression from a CMV promoter. All prime editor constructs also contained the following mutations within the MMLV RT: D200N, T306K, W313F, T330P, and L603W. All DNA repair protein and RFP expression constructs were cloned into vectors under constitutive expression from an EF1α promoter. Human MSH2, MSH6, PMS2, and MLH1 sequences were subcloned from the plasmids pFB1_hMSH2 (Addgene #129423), pFB1_hMSH6 (Addgene #129424), pFB1_PMS2 (Addgene #129425), and pFB1_MLH1 (Addgene #129426) (Geng et al., 2011). Human CDKN1A sequence was subcloned from the plasmid Flag p21 WT (Addgene #16240) (Zhou et al., 2001). Codon-optimized MLH1 sequences for human cell and mouse cell expression were designed using GenSmart Codon Optimization (Genscript) and ordered as gBlock gene fragments (Integrated DNA Technologies).

Plasmids for mammalian expression of pegRNAs or sgRNAs were cloned using Golden Gate assembly (Engler et al., 2008) as previously described (Anzalone et al., 2019). Briefly, a guide RNA vector backbone for human U6 promoter expression was digested overnight with BsaI-HFv2 (New England BioLabs) according to the manufacturer’s protocol, and linearized product was purified by electrophoresis with a 1% agarose gel using the QIAquick Gel Extraction Kit (QIAGEN). Oligonucleotides (Integrated DNA Technologies) for the spacer sequence, guide RNA scaffold, and 3′ extension were annealed, assembled with linearized U6 backbone DNA using T4 DNA ligase (New England BioLabs) according to the manufacturer’s protocol, and transformed into cells. Only guide RNA scaffold oligonucleotides were purchased with 5′ phosphorylation modifications. Some plasmids encoding pegRNAs and epegRNAs were synthesized by Twist Bioscience. A list of pegRNAs and nicking sgRNAs used in this work is provided in Table S3.

Unless otherwise noted, assembled plasmids were transformed into One Shot Mach1 cells (Thermo Fisher Scientific) and grown on Luria-Bertani (LB) or 2 × YT agar with 50 μg ml^-1^ carbenicillin (Gold Biotechnology). Plasmid sequences were fully verified by Sanger sequencing (Quintara Biosciences), and bacteria containing verified plasmids were grown in 2 × YT medium with 100 μg ml^-1^ carbenicillin (Gold Biotechnology). Plasmid DNA were isolated using the QIAGEN Plasmid Plus Midi Kit or QIAGEN Plasmid Plus Maxi Kit with endotoxin removal and 2 × the recommended amount of RNase A in Buffer P1. Some pegRNA and sgRNA plasmid DNA were isolated with the PureYield Plasmid Miniprep System (Promega Corporation) with endotoxin removal. Plasmid DNA purified using the PureYield Plasmid Miniprep System were only used for HEK293T and HeLa cell transfections. All plasmids were eluted in nuclease-free water (QIAGEN) and quantified using a NanoDrop One UV-Vis spectrophotometer (Thermo Fisher Scientific).

#### Lentivirus production for generating cell lines

To package lentivirus for generating stable cell lines, HEK293T cells were seeded on 6-well plates (Corning) at 7.5 × 10^5^ cells per well in DMEM supplemented with 10% FBS. At 60% confluency 16 h after seeding, cells were transfected with 12 μL Lipofectamine 2000 (Thermo Fisher Scientific) according to the manufacturer’s protocol and 1.33 μg lentiviral transfer plasmid, 0.67 μg pMD2.G (Addgene #12259), and 1 μg psPAX2 (Addgene #12260). 6 h after transfection, media was exchanged with DMEM supplemented with 10% FBS. 48 h after transfection, viral supernatant was centrifuged at 3000 *g* for 15 min to remove cellular debris, filtered through a 0.45 μm PVDF filter (Corning), and stored at −80°C.

#### Construction of HEK293T cell line with integrated modified HBB sequence

A lentiviral transfer plasmid (pAX198) was previously designed to contain the coding sequence of human *HBB* and a PuroR–T2A–BFP marker under expression from an EF1α promoter (pEF1α) (Hussmann et al., 2021). Lentivirus carrying this cassette were produced from HEK293T cells as described above. To stably integrate the *HBB* sequence, 6 × 10^5^ HEK293T cells were infected with lentivirus in 6-well plates (Corning) with DMEM supplemented with 10% FBS and 10 μg mL^-1^ polybrene (Sigma-Aldrich). BFP fluorescence was monitored daily using a CytoFLEX S Flow Cytometer (Beckman Coulter) to ensure an MOI of 0.1 and low copy number integration. Following infection for 2 days, HEK293T cells were selected in 2 μg μL^-1^ puromycin (Thermo Fisher Scientific) for 3 days and stable transduction was confirmed by measuring BFP fluorescence. The resulting cell line was used to optimize pegRNAs for prime editing. To measure editing, a 214-bp amplicon of the integrated *HBB* region was PCR amplified. Amplification of the endogenous *HBB* locus with these primers yields a differently sized 1064-bp amplicon.

#### Design and construction of HeLa cell line with CRISPRi sgRNA and prime edit target

The lentiviral transfer plasmid backbone for prime editing Repair-seq screens (pPC1000) was designed and cloned to contain a specific prime edit site and express a control *S. pyogenes* sgRNA for CRISPRi (Figure S1C). The prime edit site consisted of an *HBB* target for Sa-pegRNA flanked by two complementary-strand Sa-sgRNA targets derived from the *Saccharomyces cerevisiae* genome. These target sites were situated such that SaPE2–sgRNA complexes nick 50-bp upstream and 50-bp downstream of the nick formed by SaPE2–pegRNA. This 234-bp edit site was positioned adjacent to an *S. pyogenes* sgRNA expression cassette driven by a modified mouse U6 promoter such that an sgRNA and edit site could be amplified by PCR in the same 453-bp amplicon. The sgRNA expression cassette in pPC1000 encoded an EGFP-targeting control sgRNA (spacer, 5′–GACCAGGATGGGCACCACCC–3′) and an pEF1α–PuroR–T2A–BFP selection marker.

Lentivirus encoding the pPC1000 cassette were produced from HEK293T cells as described above. For stable integration, 2.5 × 10^5^ HeLa dCas9–BFP–KRAB cells were infected with pPC1000 lentivirus in 6-well plates (Corning) with DMEM supplemented with 10% FBS and 10 μg mL^-1^ polybrene (Sigma-Aldrich). BFP fluorescence was monitored with a CytoFLEX S Flow Cytometer (Beckman Coulter) to ensure an MOI of 0.1 and low copy integration. Following 2 days of infection, cells were selected in 2 μg μL^-1^ puromycin (Thermo Fisher Scientific) for 3 days and stable transduction was confirmed by measuring BFP fluorescence. The resulting HeLa dCas9–BFP–KRAB cell line with integrated pPC1000 sequence was used to pilot prime editing conditions, Sa-pegRNAs, and Sa-sgRNAs for Repair-seq screens.

#### Transfection of HEK293T, HeLa, HCT116, and N2A cells

Unless otherwise noted, HEK293T cells were seeded on 96-well plates (Corning) at 1.6–1.8 × 10^4^ cells per well in DMEM plus GlutaMAX supplemented with 10% FBS. Between 16 and 24 h after seeding, cells were transfected at 60%–80% confluency with 0.5 μL Lipofectamine 2000 (Thermo Fisher Scientific) according to the manufacturer’s protocol and 200 ng prime editor plasmid, 66 ng pegRNA plasmid, 22 ng sgRNA plasmid (where indicated), and 100 ng plasmid for RFP or MMR protein expression (where indicated).

For arrayed experiments, HeLa dCas9–BFP–KRAB and HeLa cells were seeded on 96-well plates (Corning) at 8 × 10^3^ cells per well in DMEM plus GlutaMAX supplemented with 10% FBS. Between 16 and 24 h after seeding, cells were transfected at 60%–80% confluency with 0.3 μL *Trans*IT-HeLa reagent (Mirus Bio) according to the manufacturer’s protocol and 56.25 ng prime editor plasmid bearing a P2A–BlastR selection marker, 18.75 ng pegRNA plasmid, 6.25 sgRNA plasmid (where indicated), and 28.1 ng human codon-optimized MLH1dn plasmid (where indicated). 24 h following transfection, 10 ng μL^-1^ blasticidin (Thermo Fisher Scientific) was added to each well to select for cells expressing prime editor.

HCT116 cells were seeded on 96-well plates (Corning) at 1.6 × 10^4^ cells per well in DMEM plus GlutaMAX supplemented with 10% FBS. Between 16 and 20 h after seeding, cells were transfected at 60%–80% confluency with 0.5 μL Lipofectamine 3000 plus 0.8 μL P3000 reagent (Thermo Fisher Scientific) according to the manufacturer’s protocol and 200 ng prime editor plasmid bearing a P2A–BlastR selection marker, 66 ng pegRNA plasmid, 22 ng sgRNA plasmid (where indicated), and 100 ng MLH1dn plasmid (where indicated). The day after transfection, media was replaced with fresh DMEM plus GlutaMAX supplemented with 10% FBS and 10 ng μL^-1^ blasticidin (Thermo Fisher Scientific) to select for cells expressing prime editor.

N2A cells were seeded on 96-well plates (Corning) at 1.6 × 10^4^ cells per well in DMEM plus GlutaMAX supplemented with 10% FBS. Between 16 and 20 h after seeding, cells were transfected at 60%–80% confluency with 0.5 μL Lipofectamine 2000 (Thermo Fisher Scientific) according to the manufacturer’s protocol and 175 ng prime editor plasmid, 50 ng pegRNA plasmid, 20 ng sgRNA plasmid (where indicated), and 87.5 ng plasmid encoding human codon-optimized hMLH1dn or mouse codon-optimized mMLH1dn where indicated. Genomic DNA was extracted 72 h following transfection.

#### Electroporation of HAP1, K562, and U2OS cells

HAP1 cells were electroporated using the SE Cell Line 4D-Nucleofector X Kit S (Lonza) according to the manufacturer’s protocol with 4 × 10^5^ cells (program DZ-113), 300 ng PE2–P2A–BSD, 100 ng pegRNA plasmid, and 33 ng sgRNA plasmid (where indicated). After electroporation, cells were cultured in 48-well plates (Corning) with IMDM plus GlutaMAX supplemented with 10% FBS. The day after electroporation, media was replaced with fresh IMDM plus GlutaMAX supplemented with 10% FBS and 10 ng μL^-1^ blasticidin (Thermo Fisher Scientific) to select for cells expressing prime editor.

K562 cells were electroporated using the SF Cell Line 4D-Nucleofector X Kit S (Lonza) according to the manufacturer’s protocol with 5 × 10^5^ cells (program FF-120), 800 ng prime editor plasmid, 200 ng pegRNA plasmid, 83 ng sgRNA plasmid (where indicated), and 400 ng MLH1dn plasmid (where indicated). After electroporation, cells were cultured in 6-well plates (Corning) with RPMI 1640 medium supplemented with 10% FBS and 292 μg mL^-1^ L-Glutamine (Corning).

U2OS cells were electroporated using the SE Cell Line 4D-Nucleofector X Kit S (Lonza) according to the manufacturer’s protocol with 2 × 10^5^ cells (program DN-100), 1600 ng PE2 or PE2–P2A–MLH1dn plasmid, 400 ng pegRNA plasmid, and 166 ng sgRNA plasmid (where indicated). After electroporation, cells were cultured in 12-well or 24-well plates (Greiner Bio-One) with McCoy’s 5A medium supplemented with 10% FBS.

#### Genomic DNA extraction

Unless otherwise noted, HEK293T, HeLa dCas9–BFP–KRAB, HeLa, HCT116, N2A, HAP1, K562, and U2OS cells were cultured for 72 h after transfection or electroporation before genomic DNA was isolated. Cells were washed once with PBS (Thermo Fisher Scientific) and lysed with gDNA lysis buffer (10 mM Tris-HCl, pH 8.0; 0.05% SDS; 800 units μL^-1^ proteinase K (New England BioLabs)) at 37°C for 1.5–2 h, followed by enzyme inactivation at 80°C for 30 min.

#### High-throughput amplicon sequencing of genomic DNA samples

To assess gene editing, loci were amplified from genomic DNA samples via two rounds of PCR then deep sequenced. Briefly, an initial PCR step (PCR1) amplified the genomic sequence of interest using primers (Integrated DNA Technologies) containing Illumina forward and reverse adapters. Each 20 μL PCR1 reaction was performed with 500 nM of each primer, 0.8 to 1.0 μL genomic DNA, 1 × SYBR Green (Thermo Fisher Scientific), and 10 μL Q5 High-Fidelity 2 × Master Mix (New England BioLabs) on a CFX96 Touch Real-Time PCR Detection System (Bio-Rad Laboratories) with the following thermocycling conditions: 98°C for 2 min, 29–31 cycles of [98°C for 10 s, 61°C for 20 s, and 72°C for 30 s], followed by 72°C for 2 min. PCR1 reactions were monitored with SYBR Green fluorescence to avoid over-amplification. A list of primers used for PCR1 reactions is provided in Table S4, and a list of PCR1 amplicon sequences is provided in Table S5. The subsequent PCR step (PCR2) added unique i7 and i5 Illumina barcode combinations to both ends of the PCR1 DNA fragment to enable sample demultiplexing. Each 12.5 μL PCR2 reaction was performed with 500 nM of each barcoding primer, 0.5 μL PCR1 product, and 6.25 μL Phusion U Green Multiplex PCR Master Mix (Thermo Fisher Scientific) with the following thermocycling conditions: 98°C for 2 min, 9 cycles of [98°C for 15 s, 61°C for 20 s, and 72°C for 30 s], followed by 72°C for 2 min. PCR2 products were pooled by common amplicons, separated by electrophoresis on a 1% agarose gel, purified using the QIAquick Gel Extraction Kit (QIAGEN), and eluted in nuclease-free water. DNA amplicon libraries were quantified with a Qubit 3.0 Fluorometer (Thermo Fisher Scientific), then sequenced using the MiSeq Reagent Kit v2 or MiSeq Reagent Micro Kit v2 (Illumina), with 280–300 single-read cycles. A list of FASTQ sequencing files generated in this work is provided in Table S3.

#### Quantification of amplicon sequencing data

All arrayed prime editing experiments from Figures 3, 4, 5, 6, 7, S1, S3F, S4, S5, S6, and S7 were analyzed as follows. Sequencing reads were demultiplexed using MiSeq Reporter (Illumina). Amplicon sequences (Table S5) were aligned to a reference sequence with CRISPResso2 (Clement et al., 2019) in standard mode using the parameters “-q 30” and “-discard_indel_reads TRUE.” For each amplicon, the CRISPResso2 quantification window was positioned to include the entire sequence between pegRNA- and sgRNA-directed Cas9 cut sites, as well as an additional ≥ 10 nt beyond both cut sites. For each amplicon, the same quantification window was used for PE2, PE3, PE4, and PE5 conditions, regardless of whether a nicking sgRNA was transfected. All prime editing efficiencies describe percentage of (number of reads with the intended edit that do not contain indels)/(number of reads that align to the amplicon). Single-base substitution prime editing frequencies were quantified as: (frequency of intended base substitution in reference-aligned, non-discarded reads) × (number of reference-aligned, non-discarded reads)/(number of reference-aligned reads). For all other prime edits (insertion, deletion, contiguous substitutions, combinations of edits), CRISPResso2 was run in HDR mode with all the same parameters described above and using the intended editing outcome as the expected allele (-e). Frequencies for these edits were quantified as: (number of HDR-aligned reads)/(number of reference-aligned reads). All indel frequencies were quantified as: (number of indel-containing reads)/(number of reference-aligned reads).

#### CRISPRi library cloning and lentiviral library production

An oligonucleotide library of CRISPRi sgRNAs (Q-15620 = AX227) was designed to contain 60 non-targeting control sgRNAs and 1,513 sgRNAs that target 476 genes involved in DNA repair and associated processes (Hussmann et al., 2021). A list of targeted genes and sequences in the sgRNA library is provided in Table S1. The oligonucleotide library was ordered from Twist Bioscience and sequences were amplified by PCR using Phusion High-Fidelity DNA Polymerase (Thermo Fisher Scientific) and purified with the NucleoSpin Gel and PCR Clean-up kit (Macherey-Nagel). The amplified sequences and the pPC1000 lentiviral screen vector containing the pre-validated prime edit site were digested with BstXI and BlpI restriction endonucleases (Thermo Fisher Scientific), ligated with T4 ligase (New England BioLabs), and transformed into MegaX DH10B electrocompetent cells (Thermo Fisher Scientific). The plasmid library was isolated from transformed cells using ZymoPURE II Plasmid Maxiprep Kit (Zymo Research), and the pooled library of plasmids was verified by PCR and sequencing on a MiSeq Reagent Kit v2 (Illumina).

To produce lentivirus with pPC1000 libraries, HEK293T cells were seeded in a 15 cm dish with DMEM supplemented with 10% FBS. One day after seeding, cells were transfected using 60 μL *Trans*IT-LT1 reagent (Mirus Bio) with 15 μg pPC1000 plasmid library and 5 μg packaging plasmids for expression of HIV-1 gag/pol, rev, tat, and VSV-G envelope protein. 24 h after transfection, 40 μL ViralBoost reagent (ALSTEM) was added to each 15 cm dish. 48 h after transfection, viral supernatant was collected, filtered through a 0.45 μm PVDF filter (Corning), and stored at −80°C.

#### Repair-seq screens in HeLa cells

PE2, PE3+50, PE3–50 Repair-seq screens were performed in duplicate in HeLa cells with integrated dCas9–BFP–KRAB (Addgene #46911) (Gilbert et al., 2013). These HeLa CRISPRi cells were transduced with the lentiviral library at 0.1 MOI (10.8% BFP+) in DMEM supplemented with 10% FBS, 100 U mL^-1^ penicillin, 100 μg mL^-1^ streptomycin (Thermo Fisher Scientific), and 8 μg mL^-1^ polybrene (Sigma-Aldrich). 2 days after infection, HeLa CRISPRi cells were treated with 1 μg mL^-1^ puromycin (Thermo Fisher Scientific) to select for HeLa CRISPRi cells with integrated library members. 3 days after infection, an additional 2 μg mL^-1^ puromycin was added to cells. Throughout lentiviral transduction and selection steps, cells were analyzed for BFP fluorescence on a BD LSRII flow cytometer to ensure a MOI of 0.1 and completed selection. Following 3 days of selection, media was changed to DMEM supplemented with 10% FBS, and HeLa CRISPRi cells were transfected at 50% confluency in 150 mm culture dishes (Corning) with 30 μg SaPE2–P2A–BSD plasmid, 10 μg Sa-pegRNA plasmid for installing a +6 G⋅C to C⋅G edit at the pre-validated edit site, and 3.3 μg Sa-sgRNA plasmid for +50 or –50 complementary-strand nicking (where indicated), using 140 μL *Trans*IT-HeLa reagent (Mirus Bio) according to the manufacturer’s protocol. For an unedited HeLa control condition, cells were transfected with only 30 μg SaPE2–P2A–BSD plasmid as described above. 24 h following transfection, cells were treated with 10 μg mL^-1^ blasticidin (Thermo Fisher Scientific) to select for expression of SaPE2 protein. 72 h after transfection, HeLa CRISPRi cells were washed with PBS (Thermo Fisher Scientific), resuspended using Trypsin and DMEM, and pelleted at 1000 *g* for 10 min. Finally, cells were washed once more with PBS, pelleted at 1000 *g* for 10 min, then stored at −80°C. The number of live cells collected from each Repair-seq condition is listed in Table S6.

#### Repair-seq screens in K562 cells

PE2 and PE3+50 Repair-seq screens were performed in duplicate in K562 cells with integrated dCas9–BFP–KRAB (Addgene #46911) (Gilbert et al., 2014). Cells were transduced with the lentiviral library at 0.2 MOI (18% BFP+) in RPMI supplemented with 10% FBS, 100 U mL^-1^ penicillin, 100 μg mL^-1^ streptomycin, 292 μg mL^-1^ L-Glutamine, and 8 μg mL^-1^ polybrene (Sigma-Aldrich) by centrifugation at 1000 *g* for 2 h at room temperature. 2 days post infection, cells were treated with 3 μg mL^-1^ puromycin (Gold Biotechnology) to select for cells with integrated library members. After infection, the density of the K562 CRISPRi cells was maintained at approximately 5 × 10^5^ mL^-1^ and the media was replaced with fresh RPMI supplemented with 10% FBS, 100 U mL^-1^ penicillin, 100 μg mL^-1^ streptomycin, 292 μg mL^-1^ L-Glutamine, and 3 μg mL^-1^ puromycin 3 days and 5 days post infection. During media replacement, the cells were pelleted, washed with DPBS and resuspended in fresh media to remove dead cells. All centrifugations were performed at 150 *g* for 5 min in 50 mL canonical tubes. 6 days post infection, the media was replaced twice by fresh RPMI supplemented with 10% FBS and 292 μg mL^-1^ L-Glutamine (Corning) to remove dead cells and antibiotics. Throughout lentiviral transduction and selection steps, cells were analyzed for BFP fluorescence on an Attune NxT flow cytometer to ensure completed selection. 7 days post infection, the cells were electroporated using the SE Cell Line 4D-Nucleofector X kit L (Lonza) with 1 × 10^7^ cells (program FF-120), 7.5 μg SaPE2 plasmid, 2.5 μg Sa-pegRNA plasmid for installing a +6 G⋅C to C⋅G edit at the pre-validated edit site, and 833 ng Sa-sgRNA plasmid for +50 complementary-strand nicking (for PE3+50 conditions). For an unedited K562 control condition, cells were mock electroporated without any DNA plasmid as described above. After electroporation, the cells were seeded at a density of 5 × 10^5^ mL^-1^ in RPMI supplemented with 10% FBS and 292 μg mL^-1^ L-Glutamine. 48 h post electroporation, cultures were pipetted up and down 5 times to prevent cells from clumping. 84 h post electroporation, the cells were pelleted at 1000 *g* for 10 min, washed with DPBS (Thermo Fisher Scientific), pelleted at 1000 *g* for 10 min, and then stored at −80°C. The number of live cells collected from each Repair-seq condition is listed in Table S6.

#### High-throughput sequencing of Repair-seq libraries

Genomic DNA was extracted from all Repair-seq screen cells using NucleoSpin Blood XL Maxi kit (Machery-Nagel). The entirety of the genomic DNA from each screen condition was used in the initial round of PCR (PCR1) to amplify the 453-bp region containing CRISPRi sgRNA and edit site. Each 100 μL PCR1 reaction was performed with 10 μg of genomic DNA as template, 1 μM of each primer for amplifying pPC1000 sgRNA and edit site (Table S4), and 50 μL of NEBNext Ultra II Q5 Master Mix (New England BioLabs) on a BioRad C1000 thermal cycler with the following thermocycler conditions: 98°C for 30 s, 22 cycles of [98°C for 10 s, 65°C for 75 s], followed by 65°C for 5 min. Amplification reactions were verified by TBE or agarose gel electrophoresis and ethidium bromide staining. For screens in HeLa cells, 1 mL of PCR1 product from each test condition and 1.5 mL of PCR1 product from each control condition were purified using SPRIselect (Beckman Coulter) with a double 0.5 × right side selection and a 1 × left side selection. The eluate was further purified with an additional 0.65 × left side selection using SPRIselect. For screens in K562 cells, 250 μL of PCR1 product from each condition was purified using SPRIselect with a 0.8 × left side selection. For screens in both HeLa and K562 cells, purified PCR1 amplicons were quantified using a high sensitivity DNA chip (Agilent Technologies) on an Agilent 2100 Bioanalyzer. A following PCR step (PCR2) enabled indexing of the samples by the addition of i7 and i5 Illumina barcodes, and 4 50 μL PCR2 reactions were performed for each screen condition. For each PCR2 reaction, 10 ng of PCR1 product was used as a template along with 25 μL of KAPA HiFi HotStart ReadyMix (Roche Molecular Systems) and 600 nM of each barcoding primer on a ProFlex PCR System (Applied Biosystems) with the following thermocycler conditions: 95°C for 3 min, 8 cycles of [98°C for 20 s, 65°C for 15 s, 72°C for 15 s], followed by 72°C for 1 min. The reactions were verified by TBE or agarose gel electrophoresis and ethidium bromide staining. For screens in HeLa cells, PCR2 products were purified using SPRIselect with a 0.65 × left side selection and quantified on an Agilent 2100 Bioanalyzer prior to pooling. For screens in K562 cells, PCR2 products were purified using SPRIselect with a 0.8 × left side selection. Repair-seq libraries were sequenced with the NovaSeq 6000 S1 Reagent Kit v1.5 (Illumina) with two 8-nt index reads, 44 cycles for R1 read, 263 cycles for R2 read. The number of sequencing reads acquired for each screen condition and replicate are listed in Table S6.

#### Processing of Repair-seq screen data

Repair-seq screen data was processed using a modified version of the analysis approach described in (Hussmann et al., 2021), with modifications made to accommodate the different library preparation strategy used in this study (direct amplification of genomic DNA without ligation of UMIs before amplification) and the qualitatively different categories of repair outcomes empirically observed in prime editing data.

Briefly, sequencing data for a batch of screens consists of 4 reads per cluster: 2 8-nt index reads, a 44-nt R1 read of the CRISPRi sgRNA, and a 263-nt R2 read of the repair outcome. Reads from a batch of screens are demultiplexed into individual screens based on index reads. Within each screen, reads are demultiplexed into sets representing outcomes from cells receiving each individual CRISPRi sgRNA by comparing R1 sequences to a table of expected CRISPRi sgRNAs, allowing up to one mismatch between observed and expected sequences. Because direct amplification without UMIs does not allow consensus error correction of multiple reads of each repair outcome, analysis must account for presence of errors in outcome sequences introduced by PCR or by sequencing to avoid interpreting such errors as genuine editing outcomes. As an initial triage, reads with less than 60% of base calls with a quality score greater than or equal to 30 were discarded.

To categorize a repair outcome sequencing read that passed this quality filter, the outcome was first locally aligned to the screen vector, the pegRNA sequence, the human genome (hg19) and the bos taurus genome (bosTau7) to identify a comprehensive set of alignments between portions of the outcome sequence and any of these reference sequences. The set of local alignments identified was then pruned to a parsimonious set of alignments that explains as much of the read as possible using the minimum number of alignments using a greedy approach. The parsimonious alignments are then parsed through a decision tree that examines their configuration to assign the outcome to a category.

Outcomes were classified as unedited if they consisted of a single alignment to the screen vector that did not contain the programmed SNV or any indels, with the exception of deletions of 1 nt that did not fall within 5 nt of a programmed nick, which were considered possible sequencing or PCR errors and were disregarded. Outcomes were classified as deletions if they consisted of two alignments to the screen vector that collectively covered the entire read but omitted a segment of the screen vector. Outcomes were classified as tandem duplications if they consisted of two or more alignments to the screen vector that collectively covered the entire read such that the portions of the screen vector covered by any two consecutive alignments on the read overlapped. Outcomes were classified as joining of pegRNA sequence at an unintended location if the set of parsimonious alignments included an alignment to the pegRNA such that the primer binding site (PBS) of the pegRNA was aligned to the same part of the read as the PBS in a screen vector alignment but the reverse transcription template (RTT) of the pegRNA was not aligned to the same part of the read as the RTT in a screen vector alignment. Note that in some such cases, the sequence produced is also consistent in theory with a multi-stage editing event consisting of an initial deletion or duplication that does not disrupt the PAM or protospacer followed by pegRNA-dependent editing of the resulting modified target sequence. Outcomes were classified as installation of additional edits from nearly matched scaffold sequence if the set of parsimonious alignments included an alignment to the pegRNA such that both the PBS and RTT were aligned to the same parts of the read as the PBS and RTT in a single alignment to the screen vector that covered the whole read and that the pegRNA alignment contained fewer edits relative to the outcome than the screen vector alignment.

#### Quantification of CRISPRi-induced changes in outcome frequencies

Following categorization of all outcomes for all CRISPRi sgRNAs, counts of each category for each sgRNA are collected into a matrix for downstream analysis, and the total frequency of each category across all outcomes from cells receiving non-targeting sgRNAs is calculated to establish unperturbed baseline frequencies. Because not all CRISPRi sgRNAs achieve high levels of knockdown, calculation of gene-level effects of CRISPRi sgRNAs on outcome categories must strike a balance between assigning increased confidence to phenotypes that are supported by multiple sgRNAs per gene without penalizing genes if not all sgRNAs targeting the gene have high activity. To do this, gene-level changes in outcome category frequencies in a given screen replicate (used in Figures 1E–1G, 2E–2H, and S2A–S2I) are calculated by first computing the log_2_ fold change in frequency of the category for every targeting sgRNA relative to the combined frequency across all non-targeting sgRNAs. For each gene, the gene-level log_2_ fold change is then taken to be the mean of these values for the two sgRNAs targeting the gene with the most extreme absolute values. To provide a qualitative estimate of the range of values produced in the absence of genuine signal by this process of selecting extreme values, the sixty non-targeting sgRNAs were randomly partitioned into 20 sets of 3 quasi-genes and the same process was applied to these quasi-genes.

#### Quantification of deletion boundaries

To maximize signal to noise in calculation of position-specific profiles of deletion frequencies and in relative fraction of deletions that removed sequence far outside of programmed nicks, outcomes from all sgRNAs targeting each gene were grouped together. In each such group of outcomes, an array of counts for every position in the screen vector was initialized to 0. For each read classified as a deletion, an interval from the first position deleted to the last position deleted was incremented by 1 in the array of counts. The final array of counts was then divided by the total number of outcomes. Deletions flanked by microhomology result in sequence outcomes that are consistent with two or more degenerate pairs of deletion boundaries. For these deletions, the pair with the minimum values in the coordinate system of the screen vector was arbitrarily chosen. To prevent primer dimers or other non-specific amplification products from being incorrectly identified as long deletions, apparent deletions for which the deleted region overlapped a window of 10-nt around either amplicon primer were excluded from calculation of deletion boundary statistics.

#### Design of recoded Sa-pegRNA scaffold

In Repair-seq screens performed, we observed an unintended editing outcome in which additional edits are installed from nearly matched scaffold sequence (Figures 2C, 2E, 2F, S2B–S2D, and S2G). This outcome contains a +17 T⋅A-to-C⋅G and +19 C⋅G insertion, in addition to the intended +6 G⋅C-to-C⋅G transversion (Figures 2C and S3A). These unintended edits are consistent with incorporation of an extended 3′ DNA flap generated from reverse transcription of the Sa-pegRNA scaffold sequence into the genome. Because this extended 3′ flap shares 5 nt of homology (5′–GCCAA–3′) with the genomic target sequence after the last edited nucleotide (Figure S3A), we hypothesized that disrupting this homology could reduce the frequency of incorporating these unintended edits from reverse transcription of the Sa-pegRNA scaffold. We therefore designed a recoded Sa-pegRNA that alters two base pairs within the Sa-pegRNA scaffold while preserving the same base pairing interactions (Figure S3B). The extended 3′ flap templated by this recoded Sa-pegRNA has reduced homology with the genomic target sequence. The spacer, PBS, and RT template sequences of the recoded Sa-pegRNA are identical to those for the Sa-pegRNA used in Repair-seq screens. We observed that prime editing with this recoded Sa-pegRNA mediates similar frequencies of intended editing but substantially reduced unintended scaffold sequence incorporation compared to the original Sa-pegRNA used in Repair-seq screens (Figures S3A and S3B).

#### HEK293T siRNA transfection

For experiments in Figure 3C, HEK293T cells were seeded on 6-well plates (Corning) at 7.5 × 10^5^ cells per well in DMEM plus GlutaMAX supplemented with 10% FBS. At 60% confluency 16 h after seeding, cells were transfected with 9 μL Lipofectamine RNAiMAX (Thermo Fisher Scientific) according to the manufacturer’s protocol and 90 pmol ON-TARGETplus SMARTpool siRNAs (Horizon Discovery). One day after transfection, media was replaced with fresh DMEM plus GlutaMAX supplemented with 10% FBS. 2 days after transfection, cells were washed once with PBS and resuspended using TrypLE (Thermo Fisher Scientific) and DMEM plus GlutaMAX supplemented with 10% FBS. HEK293T cells were then seeded on 96-well plates (Corning) at 2.5 × 10^4^ cells per well. Between 16 and 24 h after seeding, cells were transfected at 60%–80% confluency with 0.5 μL Lipofectamine 2000 (Thermo Fisher Scientific) according to the manufacturer’s protocol and 200 ng prime editor plasmid, 66 ng pegRNA plasmid, 22 ng sgRNA plasmid (where indicated), and 5 pmol of the same ON-TARGETplus SMARTpool siRNAs used in the first transfection. For control conditions, cells were treated with non-targeting siRNAs in both transfections. For experiments in Figure S3F, only the second transfection with PE components and siRNA was performed. Cells were cultured for 72 h after the second transfection before genomic DNA extraction.

#### Real time quantitative PCR

To measure RNAi knockdown (Figure S3E), RNA was isolated from HEK293T cells 72 h after the second siRNA transfection and converted to cDNA using the SYBR Green Fast Advanced Cells-to-CT Kit (Thermo Fisher Scientific) with cell lysis for 10-15 min using lysis solution containing 1:50 DNaseI to fully digest genomic DNA. All other steps were carried out according to the manufacturer’s protocol. Each 20 μL qPCR reaction was performed in technical and biological triplicate with 500 nM of each primer, 2 μL cDNA, 1 × SYBR Green (Thermo Fisher Scientific), and 10 μL Q5 High-Fidelity 2 × Master Mix (New England BioLabs) on a CFX96 Touch Real-Time PCR Detection System (Bio-Rad Laboratories) with the following thermocycling conditions: 98°C for 2 min, and 40 cycles of [98°C for 15 s, 65°C for 20 s, and 72°C for 30 s]. β-actin (*ACTB*) served as a housekeeping gene to normalize the amount of cDNA in each qPCR reaction. Relative RNA abundances from gene knockdown were calculated in comparison to a non-targeting siRNA control by the 2^-ΔΔCT^ method. A list of primers used for qPCR reactions is provided in Table S4.

#### Plasmid transfection dose titration in HEK293T cells

For experiments in Figure S4B, HEK293T cells were seeded on 96-well plates (Corning) at 1.6–1.8 × 10^4^ cells per well in DMEM plus GlutaMAX supplemented with 10% FBS. Between 16 and 24 h after seeding, cells were transfected at 60%–80% confluency with 0.5 μL Lipofectamine 2000 (Thermo Fisher Scientific) according to the manufacturer’s protocol and 66 ng pegRNA plasmid, 0–200 ng PE2 plasmid, and 0–100 ng for MLH1dn or RFP plasmid. Empty pUC19 filler plasmid was combined with PE2, MLH1dn, and RFP plasmids in different amounts to maintain a constant amount of total plasmid transfected (366 ng). In titrations varying the amount of total editor and *in trans* protein together, PE2 plasmid was used at a mass ratio of 2:1 with MLH1dn or RFP plasmid. Genomic DNA was isolated from cells 72 h after transfection.

#### Generation of MLH1 knock-out HeLa cell clones

One clonal wild-type HeLa cell line and two clonal Δ*MLH1* HeLa lines were used to compare prime editing enhancement from MLH1dn expression versus *MLH1* knockout (Figure S4F). To generate clonal lines, HeLa cells were seeded on 6-well plates (Corning) at 2.5 × 10^5^ cells per well in DMEM plus GlutaMAX supplemented with 10% FBS. At 60% confluency 18 h after seeding, cells were transfected using 7.5 μL *Trans*IT-HeLa reagent (Mirus Bio) according to the manufacturer’s protocol with 2 μg pLX_331-Cas9 (SpCas9 with blasticidin marker, Addgene #96924) and 500 ng sgRNA plasmid (spacer, 5′– GACAGTGGTGAACCGCATCG–3′). To make clonal wild-type HeLa cells as a control, 500 ng pUC19 plasmid was transfected instead of sgRNA plasmid. 24 h following transfection, 10 ng μL^-1^ blasticidin (Thermo Fisher Scientific) was added to each well to select for cells transfected with Cas9.

4 days following transfection, cells were plated on 96-well plates at 1 cell per well with conditioned DMEM plus GlutaMAX supplemented with 10% FBS. Single clones were grown and expanded for 18 days. To verify that Δ*MLH1* cells contain biallelic *MLH1* frameshift mutations and that control cells contain the wild-type genotype, the *MLH1* locus from clonal genomic DNA was amplified and sequenced on a MiSeq (Illumina) as described above. FASTQ sequencing files of *MLH1* in HeLa clones are listed in Table S3. HeLa Δ*MLH1* clone 1 contains *MLH1* c.55_56insA and c.41_58delinsTAACTTCC alleles. HeLa Δ*MLH1* clone 2 contains *MLH1* c.55_56insA and c.20_66del alleles. All prime editing experiments with these clonal HeLa lines were performed as described above for HeLa cells.

#### Prime editing of contiguous substitutions and additional silent mutations

Seven sets of prime edits that substitute 1–5 contiguous bases (35 edits total) were tested across five loci in HEK293T cells (Figures 5E, 5F, and S5J). Within each set of contiguous substitutions, all five edits altered at least one base within the seed region of the pegRNA protospacer (+1–3 nucleotides), at least one base within the PAM sequence of the pegRNA protospacer (+5 G or +6 G), or no bases within the seed region or PAM sequence at all. Because prime edits that alter the seed region or PAM sequence are more efficiently made, the design of these contiguous substitution edits controls for these confounding effects on editing efficiency, thereby enabling comparison of editing efficiency within each set.

Six sets of prime edits that program a coding change with or without additional silent mutations (27 edits total) were tested across six gene targets in HEK293T cells (Figures 5H and S5K). Each of the six coding edits makes a transversion at one of the PAM nucleotides of the pegRNA protospacer (+5 G or +6 G). This design controls for confounding effects on editing efficiency (as explained above), allowing comparison of editing efficiency within each set. Silent mutations were designed to be close to (typically within 5 bp from) the intended coding edit to maximize interference of MMR recognition of the intended coding edit. The frequency of reads that contain the intended coding edit without indels and with or without any additional silent mutations was quantified using CRISPResso2 as described above.

#### Analysis of prime editor activity at Cas9 off-target sites

Prime editor activity at known Cas9 off-target sites was determined by sequencing genomic DNA from HEK293T cells 3 days after transfection with plasmids encoding PE2, pegRNAs, and MLH1dn (where indicated) as described above. The top 4 off-target sites for each of the *HEK3*, *EMX1*, *FANCF*, and *HEK4* spacers previously detected by circularization for *in vitro* reporting of cleavage effects by sequencing (CIRCLE-seq, Tsai et al., 2017) (16 sites total) were deep sequenced from genomic DNA samples as described above. To analyze off-target editing, reads were aligned to reference off-target amplicons using CRISPResso2 (Clement et al., 2019) in standard mode with the parameters “-q 30” and “-w 10.” Off-target reads were called as leniently as possible to capture all potential reverse transcription products. For each off-target reference amplicon, the nucleotide sequence 3′ of the Cas9 nick site (prime-editable target) was compared to the 3′ DNA flap sequence encoded by pegRNA reverse transcription. Counting from the 5′ ends, the minimum sequence of the 3′ DNA flap that deviates from prime-editable target sequence was designated as an off-target marker sequence. All reference-aligned reads that contain this off-target marker sequence directly 3′ of the Cas9 nick site (including indel-containing reads) were called as off-target reads. Off-target editing efficiencies were thus quantified as a percentage of (number of off-target reads)/(number of reference-aligned reads). We note that for some amplicons, mismatch rates at the relevant editing position were comparable to rates at other positions in the amplicon, suggesting that context-specific sequencing errors may contribute to apparent off-target prime editing and therefore this conservative approach may overestimate the true rate of pegRNA-mediated editing at off-target sites.

#### Sequencing of microsatellite instability in genomic DNA

Microsatellite instability was assessed in genomic DNA from HCT116 cells, monoclonal wild-type HAP1 cells, monoclonal HAP1 cells grown for 2 months (∼60 cell divisions) following *MSH2* knockout, and HeLa cells 3 days after transfection with plasmids encoding PE2–P2A–BSD, pegRNA, and MLH1dn where indicated. HeLa cell transfections were performed as described above. 17 mononucleotide repeats that are highly sensitive to MMR activity and are widely used to diagnosis MMR deficiency in tumors (Bacher et al., 2004; Hempelmann et al., 2015; Umar et al., 2004) were deep sequenced from genomic DNA samples. The first PCR reaction (PCR1) amplified the microsatellite sequence of interest using primers (Integrated DNA Technologies) containing Illumina forward and reverse adapters. Each 20 μL PCR1 reaction was performed with 250 nM of each primer, 0.8 μL genomic DNA, 1 × SYBR Green (Thermo Fisher Scientific), and 10 μL Q5 High-Fidelity 2 × Master Mix (New England BioLabs) on a CFX96 Touch Real-Time PCR Detection System (Bio-Rad Laboratories) with the following thermocycling conditions: 98°C for 3 min, 30 cycles of [98°C for 15 s, 62°C for 30 s, and 72°C for 30 s], followed by 72°C for 3 min. All 17 PCR1 products amplified from the same genomic DNA sample were pooled, purified with 0.8 × AMPure XP beads (Beckman Coulter), and eluted in nuclease-free water. A list of primers used for PCR1 reactions is provided in Table S4, and a list of PCR1 amplicon sequences if provided in Table S5. The subsequent PCR step (PCR2) added unique i7 and i5 Illumina barcode combinations to both ends of the PCR1 DNA amplicons to enable sample demultiplexing. Each 20 μL PCR2 reaction was performed with 500 nM of each barcoding primer, 25 ng of pooled PCR1 product, 1 × SYBR Green, and 10 μL Q5 High-Fidelity 2 × Master Mix on a CFX96 Touch Real-time PCR Detection System with the following thermocycling conditions: 98°C for 2 min, 8 cycles of [98°C for 15 s, 61°C for 20 s, and 72°C for 30 s], followed by 72°C for 2 min. All PCR2 products were pooled, purified with 0.8 × AMPure XP beads, and eluted in nuclease-free water. DNA amplicon libraries were quantified with a Qubit 3.0 Fluorometer (Thermo Fisher Scientific), then sequenced using the MiSeq Reagent Kit v2 (Illumina) with 300 single-read cycles. A list of FASTQ sequencing files generated in these experiments is provided in Table S3.

#### Quantification of microsatellite instability

The 17 microsatellites analyzed all consist of long homopolymers. To quantify the observed lengths of these microsatellites in a way that is robust against the high rate of sequencing errors observed in homopolymers, we searched each sequencing read for the sequences expected to flank the homopolyers and then considered the final length of the homopolymer to be the distance between these flanking sequences. Specifically, for each locus, the longest homopolymer within the amplicon was identified, and 20-nt of the expected reference sequence on either side was recorded. Sequencing reads were demultiplexed into their loci of origin based on the first 20-nt of each read. Within reads for each locus, for each sequencing read, the first occurrences of sequences within Hamming distance 2 of the two flanking sequences were recorded. If both flanking sequences were located in the expected relative orientation within 50-nt of each, the distance between was recorded.

#### Prime editing of therapeutically relevant loci

To demonstrate the applicability of PEmax and PE4 and PE5 systems, we tested prime editing at six disease-relevant sites (Figure 7D). First, we made a silent G⋅C-to-A⋅T transversion at the 6^th^ codon of *HBB*, which is mutated in sickle cell disease patients (Ingram, 1956). Second, we installed the G127V allele (a G⋅C-to-T⋅A transversion) in *PRNP* that confers resistance to prion disease (Asante et al., 2015; Mead et al., 2009). Third, we introduced a silent C⋅G-to-T⋅A mutation at a *CDKL5* site known to contain a causative mutation for CDKL5 deficiency disorder, a severe neurodevelopmental condition (Olson et al., 2019). Fourth, we installed the *CXCR4* P191A allele (a G⋅C-to-C⋅G edit) that inhibits HIV infection in human cells (Liu et al., 2018). Fifth, we generated the *IL2RB* H134D Y135F (non-adjacent T⋅A-to-A⋅T and G⋅C-to-C⋅G edits) variant that enables orthogonal IL-2 receptor responsiveness for adoptive T cell transfer therapy (Sockolosky et al., 2018). Lastly, we recoded the BCL11A repressor binding site within the *HBG1* and *HBG2* fetal hemoglobin gene promoters to a GATA1 transcriptional activator motif (non-adjacent G⋅C-to-A⋅T and C⋅G-to-A⋅T edits), which in principle could induce fetal hemoglobin expression for treatment of hemoglobinopathies (Amato et al., 2014).

#### *In vitro* transcription of prime editor and MLH1dn mRNA used in iPSC and T cell experiments

As described previously (Nelson et al., 2021), plasmids were cloned to encode an inactivated T7 promoter followed by a 5′ untranslated region (UTR), Kozak sequence, coding sequences of PE2 or MLH1dn, and a 3′ UTR. T7 promoter inactivation prevents potential transcription from circular plasmid template during mRNA generation. These components together were PCR amplified with Phusion U Green Multiplex Master Mix (Thermo Fisher Scientific) using primers that correct T7 promoter inactivation and append a 119-nt poly(A) tail to the 3′ UTR. The resulting PCR product was purified with the QIAquick PCR Purification Kit (Thermo Fisher Scientific) and served as a template for subsequent *in vitro* transcription. PE2 and MLH1dn mRNAs were transcribed from these templates using the HiScribe T7 High-Yield RNA Synthesis Kit (New England BioLabs) with co-transcriptional capping by CleanCap AG (TriLink Biotechnologies) and full replacement of UTP with N^1^-Methylpseudouridine-5′-triphosphate (TriLink Biotechnologies). Transcribed mRNAs were precipitated in 2.5 M lithium chloride (Thermo Fisher Scientific), washed twice in 70% ethanol, then dissolved in nuclease-free water. The resulting PE2 and MLH1dn mRNA was quantified with a NanoDrop One UV-Vis spectrophotometer (Thermo Fisher Scientific) and was stored at −80°C.

#### Electroporation of human patient-derived induced pluripotent stem cells

Prior to electroporation, 24-well culture plates (Thermo Fisher Scientific) were coated with 250 μL rhLaminin-521 (Thermo Fisher Scientific) diluted 1:40 in DPBS (Thermo Fisher Scientific) per well, and incubated at 37°C in a 5% CO_2_ incubator for 2 h. For electroporation, iPS cell colonies at 70%–80% confluency were washed once with DPBS and dissociated in pre-warmed Accutase (Innovative Cell Technologies) for 10 min at 37°C in a 5% CO_2_ incubator. Next, iPS cells were gently triturated, moved into a sterile 15 mL conical tube, then combined with an equal volume of DMEM/F12 (Thermo Fisher Scientific) to quench dissociation enzyme activity. Cells were pelleted at 300 *g* for 3 min and resuspended in StemFlex medium (Thermo Fisher Scientific) supplemented with 10 μM Y-27632 (Cayman Chemical). Cell counts and viability were determined using the Countess II FL Automated Cell Counter (Thermo Fisher Scientific). For electroporation using the NEON Transfection System 10 μL kit (Thermo Fisher Scientific), 2 × 10^5^ iPS cells were pelleted at 300 *g* for 3 min and resuspended in 9 μL NEON Buffer R. The cell solution was combined with a 3 μL mixture of 1 μg PE2 mRNA, 90 pmol synthetic pegRNA (Integrated DNA Technologies), 60 pmol synthetic sgRNA (Synthego) where indicated, and 0–2 μg MLH1dn mRNA in NEON Buffer R. Synthetic pegRNAs and sgRNAs were dissolved in TE buffer (10 mM Tris-HCl, pH 8.0; 0.1 mM EDTA). Mock control electroporations were performed with 3 μL NEON Buffer R without any RNA added. Directly prior to electroporation, rhLaminin-521 was aspirated and immediately replaced with 250 μL pre-warmed StemFlex medium supplemented with 10 μM Y-27632 per 24-well. Next, 10 μL of the combined cell and RNA mixture was electroporated using the NEON Transfection System (Thermo Fisher Scientific) with the following parameters: 1400 V, 20 ms, one pulse. Cells were seeded immediately into rhLaminin-521-coated 24-well plates with 250 μL StemFlex medium supplemented with 10 μM Y-27632 per well. Media was changed the following day with 500 μL StemFlex medium supplemented with 5 μM Y-27632. 72 h following electroporation, media was changed to 500 μL StemFlex medium per well. Genomic DNA was extracted 96 h after electroporation by washing iPS cells once with DPBS, lysing with gDNA lysis buffer (10 mM Tris-HCl, pH 8.0; 0.05% SDS; 800 units μL^-1^ proteinase K (New England BioLabs)) at 37°C for 2 h, followed by enzyme inactivation at 80°C for 30 min. All iPSC electroporations were performed in technical duplicate and biological triplicate.

Following amplicon sequencing of the edited *CDKL5* locus, frequencies of intended editing and indels were quantified with CRISPResso2 in HDR mode, as described above. Because patient-derived iPSCs were heterozygous for the c.1412delA allele, the frequency of editable alleles with the intended edit was quantified as: (editing frequency – editing frequency in mock controls)/(100 – editing frequency in mock controls). Frequency of editable alleles with indels was quantified as described above: (total number of indel-containing reads)/(number of amplicon-aligned reads). The resulting frequencies of editable alleles with the intended edit or indels were averaged between technical duplicates, and values from biological triplicates are shown.

#### Electroporation of primary human T cells

Prior to electroporation, T cells were activated for 2 days with Dynabeads Human T-Expander CD3/CD28 (Thermo Fisher Scientific) and cultured at 37°C and 5% CO_2_ in T cell media (X-VIVO 15 Serum-free Hematopoietic Cell Medium (Lonza), supplemented with 5% AB human serum (Valley Biomedical), 1 × GlutaMAX (Thermo Fisher Scientific), 12 mM N-acetyl-cysteine (Sigma Aldrich), 50 U mL^-1^ penicillin and 50 μg mL^-1^ streptomycin (Thermo Fisher Scientific), 300 IU mL^-1^ IL-2 (Peprotech), and 5 ng mL^-1^ recombinant human IL-7 (Peprotech) and IL-15 (Peprotech)). CD3/CD28 beads were removed from cells 5–7 h before electroporation. For electroporation using the NEON Transfection System 10 μL kit (Thermo Fisher Scientific), 3.0–3.5 × 10^5^ cells per sample were pelleted by centrifugation for 5 min at 300 *g* and resuspended in 11 μL NEON Buffer T. The cell solution was added to a mix of 1 μg PE2 mRNA, 90 pmol synthetic pegRNA (Integrated DNA Technologies), 60 pmol synthetic sgRNA (Synthego), and 0–2 μg MLH1dn mRNA. Synthetic pegRNAs and sgRNAs were dissolved in TE buffer (10 mM Tris-HCl, pH 8.0; 0.1 mM EDTA). Mock control electroporations were performed with 3 μL NEON Buffer T without any RNA added. Electroporation on the NEON Transfection System (Thermo Fisher Scientific) was carried out using 10 μL NEON tips with the following parameters: 1,400 V, 10 ms, three pulses. Cells were plated in 600 μL fresh T cell media in a 24-well plate. 2 days after electroporation, cell counts and viability were determined using the Countess II Automated Cell Counter (Thermo Fisher Scientific), and 1 mL fresh T cell media was added to cells. 4 days after electroporation, cells were pelleted by centrifugation for 5 min at 300 *g* and genomic DNA was isolated using the PureLink Genomic DNA Mini Kit (Thermo Fisher Scientific) following the “mammalian cells lysate” protocol with elution in nuclease-free water.

### Quantification and statistical analysis

The number of independent biological replicates and technical replicates for each experiment are described in the figure legends or STAR Methods section. In Figure 5I, a non-parametric Mann-Whitney *U* test was used to compare prime editing data in HEK293T cells with prime editing data in HeLa, K562, and U2OS cells.

## Data Availability

Amplicon sequencing data generated during this study are available at the NCBI Sequence Read Archive database under PRJNA735408. Data from Repair-seq screens are available under PRJNA734952. Processed Repair-seq screen data are available at DOI: https://doi.org/10.5281/zenodo.5551032. The code used for data processing and analysis are available at https://github.com/pinellolab/CRISPResso2 and https://github.com/jeffhussmann/repair-seq (DOI: https://doi.org/10.5281/zenodo.5534778).

## Supplemental information

Table S1. List of CRISPRi guide RNAs in prime editing Repair-seq libraries, related to Figures 1 and 2 Sequences of each CRISPRi sgRNA spacer and the associated oligonucleotide used to clone lentiviral plasmid libraries for Repair-seq screens (additional details in STAR Methods).

Table S2. Frequencies of the intended prime edit in Repair-seq screens, related to Figure 1 Percentage of reads with only the programmed +6 G⋅C-to-C⋅G prime edit and no unintended byproducts in Repair-seq screens, grouped by CRISPRi sgRNA.

Table S3. List of sequencing data and guide RNAs from all experiments, related to Figures 1, 2, 3, 4, 5, 6, and 7 List of every high-throughput sequencing experiment performed with the associated treatment conditions, sequences of pegRNA and sgRNA used, sequenced amplicons, and names of FASTQ files deposited at the NCBI Sequence Read Archive database under PRJNA735408.

Table S4. Primers used for sequencing of genomic DNA and qRT-PCR, related to Figures 1, 2, 3, 4, 5, 6, and 7

Table S5. Nucleotide sequences of amplicons from high-throughput sequencing, related to Figures 1, 2, 3, 4, 5, 6, and 7

Table S6. Number of cells and sequencing reads from Repair-seq screens, related to Figures 1 and 2 The first sheet contains the number of cells and sequencing reads recovered for each Repair-seq screen condition. The second sheet contains the number of sequencing reads recovered for each CRISPRi sgRNA in each Repair-seq screen. Unedited HeLa screen controls were transfected with SaPE2 without Sa-pegRNA. Unedited K562 screen controls were transfected without any plasmid DNA.
